# Hepsin as a potential therapeutic target for alleviating acetaminophen-induced hepatotoxicity via gap-junction regulation and oxidative stress modulation

**DOI:** 10.1007/s10565-024-09915-z

**Published:** 2024-09-18

**Authors:** Yu-Fei Tsai, Chien-Hung Chen, Yao-Ming Wu, Chia-Lu Hung, Mo-Chu Fang, I.-Shing Yu, Jin-Chuan Sheu, Yu-Chen Hsu, Shu-Wha Lin

**Affiliations:** 1 Department of Clinical Laboratory Sciences and Medical Biotechnology, College of Medicine, National Taiwan University, Taipei, Taiwan; 2 Department of Internal Medicine, National Taiwan University Hospital, College of Medicine, National Taiwan University, Taipei, Taiwan; 3 Department of Medicine, National Taiwan University Cancer Center, Taipei, Taiwan; 4 Department of Surgery, National Taiwan University Hospital, College of Medicine, National Taiwan University, Taipei, Taiwan; 5 Department of Surgical Oncology, National Taiwan University Cancer Center, Taipei, Taiwan; 6 Laboratory Animal Center, College of Medicine, National Taiwan University, Taipei, Taiwan; 7 Liver Disease Prevention and Treatment Research Foundation, Taipei, Taiwan; 8 Department of Laboratory Medicine, National Taiwan University Hospital, College of Medicine, National Taiwan University, Taipei, Taiwan

**Keywords:** Liver, Acetaminophen, Drug-induced hepatotoxicity, Gap junction, Oxidative stress, Hepsin, Type II transmembrane serine protease (TTSP)

## Abstract

Acetaminophen (APAP) overdose is a leading cause of drug-induced liver damage, highlighting the limitations of current emergency treatments that primarily involve administering the glutathione precursor N-acetylcysteine and supportive therapy. This study highlights the essential protective role of the type II transmembrane serine protease (TTSP), hepsin, in mitigating acetaminophen-induced liver injury, particularly through its regulation of gap junction (GJ) abundance in response to reactive oxygen stress in the liver. We previously reported that reduced levels of activated hepatocyte growth factor and the c-Met receptor tyrosine kinase—both of which are vital for maintaining cellular redox balance—combined with increased expression of GJ proteins in hepsin-deficient mice. Here, we show that hepsin deficiency in mice exacerbates acetaminophen toxicity compared to wild-type mice, leading to more severe liver pathology, elevated oxidative stress, and greater mortality within 6 h after exposure. Administering hepsin had a protective effect in both mouse models, reducing hepatotoxicity by modulating GJ abundance. Additionally, transcriptome analysis and a functional GJ inhibitor have highlighted hepsin's mechanism for managing oxidative stress. Combining hepsin with relatively low doses of N-acetylcysteine had a synergistic effect that was more efficacious than high-dose N-acetylcysteine alone. Our results illustrate the crucial role of hepsin in modulating the abundance of hepatic GJs and reducing oxidative stress, thereby offering early protection against acetaminophen-induced hepatotoxicity and a new, combination approach. Emerging as a promising therapeutic target, hepsin holds potential for combination therapy with N-acetylcysteine, paving the way for novel approaches in managing drug-induced liver injury.

**Graphical Abstract:**

1. Hepsin−/− mice exhibit exacerbated APAP toxicity, resulting in more severe liver damage, elevated oxidative stress, and higher mortality.

2. Hepsin is crucial in protecting against APAP-induced liver injury by regulating gap junctions and reducing oxidative stress.

3. Combining hepsin with low doses of N-acetylcysteine provides greater protection against APAP-induced hepatotoxicity than high-dose NAC alone.

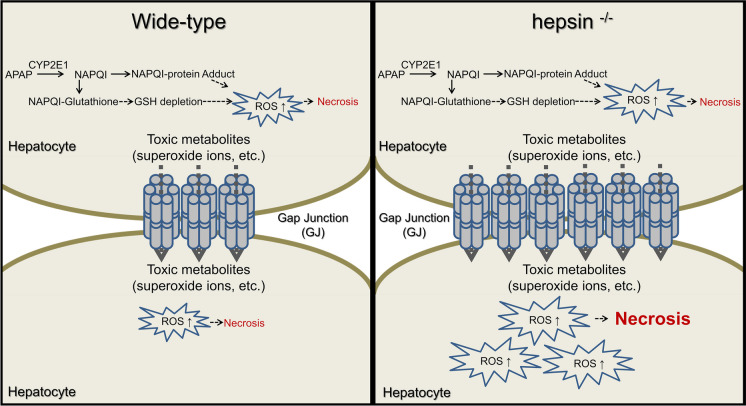

**Supplementary Information:**

The online version contains supplementary material available at 10.1007/s10565-024-09915-z.

## Introduction

Drug-induced liver injury have now gradually become the leading causes of acute liver failure in developed countries and acetaminophen (APAP) abuse is the predominant cause of drug-induced hepatotoxicity with associated acute liver failure (Fisher and Curry 2019; Hillman et al. 2016; Reuben et al. 2016). Currently, the only U.S. Food and Drug Administration-approved treatment for APAP overdose is N-acetylcysteine (NAC), a glutathione (GSH) precursor, administered alongside supportive therapy (European Association for the Study of the Liver. Electronic address et al. 2019). However, the therapeutic effectiveness of NAC for APAP overdose is limited by a narrow optimal administration window—within 8 h post-exposure—and is further challenged by the increasing incidence of treatment failures in overdose cases (Shingina et al. 2023). Due to its low bioavailability, NAC must be administered at high doses, which can disrupt hepatocyte metabolism and regenerative processes, increase the risk of adverse reactions (Ntamo et al. 2021). These adverse effects include nausea, vomiting, anaphylactoid reactions, bronchospasm, hypotension (Ershad et al. 2019; Hendrickson 2019), and can even be fatal in some cases (Chowdhury et al. 2020). Given these limitations, there is an urgent need for more effective treatments for APAP overdose.

When administered at therapeutic levels, APAP is primarily metabolized via the glucuronidation pathway, producing non-toxic metabolites that the liver and kidneys can further process (Chiew and Isbister 2023). Excessive amounts of APAP metabolism in the liver generate a toxic intermediate, N-acetyl-p-benzoquinone imine (NAPQI), mainly through the activity of cytochrome P450 enzymes CYP2E1 and CYP1A2. NAPQI, characterized by its reactive quinone imine group, is prone to redox reactions and may lead to the generation of reactive oxygen species (ROS). Normally, NAPQI is efficiently scavenged by hepatic GSH stores in hepatocytes. Once GSH is depleted, ROS and reactive nitrogen species escalate, leading to increased oxidative stress, mitochondrial dysfunction, and DNA damage; NAPQI further exacerbates this by binding to certain intracellular proteins, disrupting their functions and causing cellular damage (Ramachandran et al. 2011). Several complex signal transduction pathways and cellular processes have been implicated in APAP hepatotoxicity, including those involving c-Jun N-terminal kinase, Nuclear factor E2-related factor 2 (Nrf2), p53, endoplasmic reticulum stress, autophagy, and sterile inflammation (Chowdhury et al. 2020; Yan et al. 2018). Despite this, a comprehensive understanding of these pathways is lacking, driving the search for more effective management strategies for APAP hepatotoxicity.

Previous studies have highlighted the important role played by hepatocyte gap junctions (GJs) with regard to increasing the severity of APAP-induced liver toxicity. This is attributed to the ability of free radicals within cells to traverse GJs and enter adjacent hepatocytes. There is a notable association between the expression of connexin 32 (Cx32), which is a critical component of GJs, and the onset of APAP-induced hepatotoxicity (Patel et al. 2012). Thus, strategies to inhibit GJ function, including the deletion of the Cx32 gene (Naiki-Ito et al. 2010), impairment of its activity (Park et al. 2013), or use of small-molecule inhibitors to block its function (Patel et al. 2012), have shown effectiveness in diminishing APAP-related liver toxicity.

Hepsin is a member of the type II transmembrane serine protease (TTSP) family (Leytus et al. 1988) and is predominantly found in the liver (Tsuji et al. 1991). Research from our laboratory has revealed that hepsin functions as an activator of the precursor of hepatocyte growth factor (HGF), thereby influencing the expression of hepatocellular GJ proteins via the HGF-cMet pathway. In hepsin-deficient (hepsin^−/−^) mice, a notable inhibition of HGF-cMet signaling was observed, characterized by decreased levels of activated HGF and reduced cellular abundance of phosphorylated/activated cMet. This inhibition resulted in a two-fold increase in GJ expression in the liver compared to wild-type mice (Hsu et al. 2012). Importantly, HGF-cMet signaling is crucial for maintaining cellular redox balance, with HGF acting as an antioxidant and c-Met signaling crucial to limiting the overproduction of endogenous ROS (Gloire et al. 2006; Kannan et al. 2004). Given the reduced levels of activated HGF and cMet in hepsin^−/−^ mice, combined with the increased abundance of GJs, we hypothesize that these mice may be more vulnerable to oxidative stress, potentially exacerbating the severity of APAP-induced liver injury.

This study highlights the protective role of hepsin in APAP-induced liver injury. The absence of hepsin, as in hepsin^−/−^ mice, resulted in increased APAP toxicity, leading to decreased overall survival and accelerated mortality. This was further substantiated by the observation that inhibiting heightened GJ function with a GJ inhibitor in mice effectively diminished APAP toxicity, which correlated with reduced levels of oxidative stress in hepatocytes. The synergistic effect of combining hepsin with clinically low doses of NAC appeared to be more effective than the standard clinical care of high-dose NAC for APAP overdose. Overall, our results propose a novel therapeutic strategy for APAP-induced liver injury, involving hepsin administration alongside a low dose of NAC to improve treatment effectiveness and address current limitations.

## Materials and methods

### Animals

The hepsin^−/−^ mice were described before (Yu et al. 2000). The hepsin^−/−^ mice were backcrossed to C57BL/6JNarl mice for more than 10 generations. Adult male 8–12 week-old hepsin^−/−^ and wild-type mice, obtained from heterozygous inbreeding, were used throughout the study (Fig. S1). Mice were fasted for 16 to 18 h before receiving APAP by intraperitoneal injection (Hu et al. 2024; Kim et al. 2018) and were sacrificed at time points according to the experimental design. All animal experiments were approved by the Board of Animal Welfare of National Taiwan University College of Medicine and performed according to its guidelines (IACUC No. 20200110, IACUC No. 20220389).

### Plasmid for single-stranded adeno-associated viral vectors 2/8

The plasmid pAAV-MCS-hAAT/EGFP was designed for maximum transgene expression and includes the hepatic locus control region from the ApoE gene (ApoE-HCR), a liver-specific α1-antitrypsin promoter (hAAT promoter), coagulation factor IX intron A (FIX intron A), a 3' mRNA transcription termination/polyadenylation signal (bghpA), and a reporter gene encoding enhanced green fluorescent protein (EGFP), as previously described (Miao et al. 2001). The cDNA sequences for wild-type human hepsin (hHPN^WT^) and a loss-of-function mutant (hHPN^RS^; R162A and S353Y double mutant) were used to replace the EGFP gene, generating the AAV-hHPN^WT^ and AAV-hHPN^RS^ vectors. AAV-EGFP served as the vector control in this study. The recombinant AAVs were produced and packaged by the AAV Core Facility of Academia Sinica (Grant AS-CFII112-204).

### Overexpression of human hepsin in adult mice with AAV administration

For the APAP susceptibility test, mice were made to overexpress human hepsin in the liver through transduction with 2.5 × 10^10^ viral genome (vg) per mouse of AAV-hHPN^WT^/hHPN^RS^/EGFP via retro-orbital injection. Three weeks after AAV transduction, hepsin^−/−^ mice were administered with 400 mg/kg APAP, followed by liver function biochemistry, histological, and pathological analyses. Anticipating that wild-type mice would exhibit increased susceptibility and a more pronounced response to a higher APAP dose, AAV-transduced wild-type mice were treated with 600 mg/kg APAP for subsequent analysis.

### Treatment of mice with 2APB

2APB (Sigma, D9754) was made fresh for each experiment (dissolved in DMSO at 100 mg/ml as the stock). 2APB was dosed at 1 or 20 mg/kg and administered to mice 2 h before treatment with 500 mg/kg APAP. The body weight of adult mice was estimated at 25 g, and the intraperitoneal injection volume was 250 µl; the 0.1 mg/ml or 2 mg/ml 2PAB solution was prepared from a 100 mg/ml stock with appropriate dilution with 0.9% saline. All vehicle-control mice received the same volume of 0.1% or 2% DMSO diluted with 0.9% saline.

### Treatment of mice with NAC

NAC (Sigma, A8199) was made fresh for each experiment in 0.9% saline at 20 or 30 mg/ml. NAC was dosed at 200 or 300 mg/kg via intraperitoneal injection at 1 h after treatment with 600 mg/kg APAP.

### Statistical analyses

The Mann–Whitney test or Welch's t-test was used to assess the statistical significance of differences between values for data collected from all assays. All survival curve statistics were collected using the log-rank test. P-values of < 0.05 were considered statistically significant.

Additional information on materials and methods can be found in the Supplementary Materials and Methods.

## Results

### Increased severity of liver injury and mortality of mice with hepsin deficiency during the early period after acetaminophen exposure

**Fig. 1:**
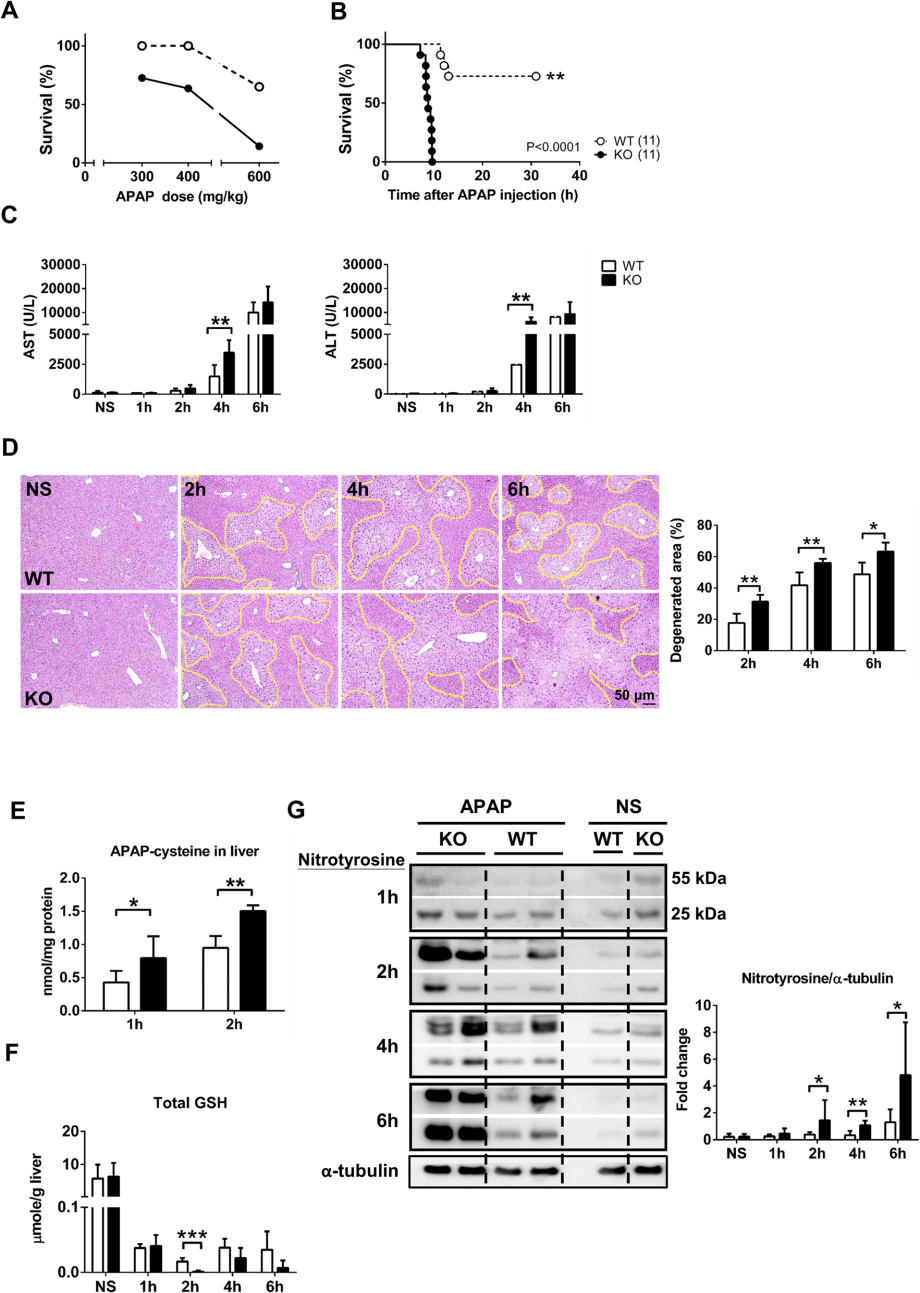
APAP induces aggravated liver injury and early lethality in hepsin^−/−^ mice. (**A**) Survival rate of wild-type (WT) and hepsin^–/–^ (KO) mice were assessed 8 h after APAP treatment at doses of 300, 400, or 600 mg/kg. (**B**) Survival rate after 400 mg/kg APAP treatment. (**C**) Measurement of serum ALT (alanine aminotransferase) and AST (aspartate aminotransferase) levels at the indicated time points after administering 400 mg/kg APAP. (**D**) Images of liver pathology, shown by hematoxylin and eosin staining and quantification of degenerated area as a percentage at the indicated time points after 400 mg/kg APAP treatment (n = 5–7 per group). (**E**) Levels of APAP-cysteine in mouse liver tissue at 1 and 2 h after 400 mg/kg APAP treatment (n = 6–8 per group). (**F**) Levels of total glutathione (GSH) in mouse liver tissue at the indicated time points after 400 mg/kg APAP treatment (n = 6–9 per group). (**G**) Western blot analysis of nitrotyrosine in mouse liver tissue at the indicated time points after 400 mg/kg APAP treatment (n = 6–10 per group). In the survival analysis, sample sizes for each group are indicated in brackets, and statistical significance was determined using the log-rank test with a *p*-value of < 0.0001. Data are presented as the mean ± SD in bar charts,with significance levels denoted by asterisks: *p < 0.05, **p < 0.01, ***p < 0.001

To investigate the potential physiological role of endogenous hepsin in the early response to APAP-induced liver injury, we analyzed hepsin expression in wild-type mouse liver lysates following APAP treatment using western blotting (Fig. S2). Hepsin levels remained stable at 0.5 h post-treatment with 400 mg/kg APAP, comparable to those in saline-treated control livers. However, a significant 70% reduction in hepsin expression was detected at the 1-h time point after APAP administration. Notably, histological analysis at this time point revealed no significant hepatocyte damage, suggesting that the rapid decrease in hepsin protein levels may primarily result from the activation and subsequent degradation of the hepsin serine protease in response to APAP-induced hepatotoxicity. Therefore, various APAP doses were tested to assess susceptibility to APAP-induced toxicity, aiming to investigate potential differences in tolerance between wild-type and hepsin^−/−^ mice. After 8 h of exposure, the hepsin^−/−^ mice exhibited lower survival rates compared with the wild-type mice at different APAP concentrations. This outcome displayed a dose-dependent negative correlation between the APAP doses and survival rate (Fig. 1A). Moreover, at an APAP dose of 400 mg/kg, hepsin^−/−^ mice experienced rapid mortality within 8 to 10 h; in contrast, more than 80% of the wild-type mice survived beyond 30 h under the same conditions (Fig. 1B). For the hepsin^−/−^ mice, the timing of mortality was such that it occurred prior the onset of liver repair and regeneration processes, thereby confirming the role of hepsin in determining the mechanism of APAP toxicity tolerance during the early phase of drug exposure.

To understand the differences in APAP-induced toxicity between hepsin^−/−^ and wild-type mice, the activities of various relevant enzymes were measured at 1, 2, 4, and 6 h after APAP administration. At the 4-h time point, serum levels of alanine transaminase and aspartate transaminase (AST) were markedly higher in the hepsin^−/−^ mice, indicating more severe liver damage compared with their wild-type counterparts (Fig. 1C). Furthermore, quantification of hepatocellular vacuolation (a characteristic of cellular degeneration observed through tissue pathology staining) at the 2-h time point revealed a 1.5-fold greater area of liver degeneration in hepsin^−/−^ mice compared with wild-type mice, which increased to ~ 2.5-fold greater by the 6-h time point (Fig. 1D). This correlation with the quantified degeneration area further supported the idea that, in the absence of hepsin, hepatic impairment is more severe during the early stages of APAP exposure.

To investigate the severe liver damage and increased mortality observed in hepsin^−/−^ mice during the early stages after APAP administration, we employed ultra-performance liquid chromatography-tandem mass spectrometry to measure levels of APAP-cysteine adducts in mouse liver tissue, which indirectly assesses the generation of the toxic APAP metabolite, NAPQI (Hairin et al. 2013). The hepsin^−/−^ mice had higher levels of APAP-cysteine adducts in the liver at 1 and 2 h post-APAP exposure compared with wild-type mice (Fig. 1E). Subsequently, we evaluated the levels of two crucial APAP-metabolizing enzymes, CYP2E1 and CYP1A2, revealing no significant differences in the protein expression levels and activities of CYP1A2 between hepsin^−/−^ and wild-type mice either before or after APAP administration (Fig. S3).

At 2 h post-APAP treatment, hepsin^−/−^ mice also exhibited a greater reduction in total GSH compared with wild-type mice (Fig. 1F), although GSH levels did not differ significantly between the hepsin^−/−^ and wild-type controls treated with normal saline. Moreover, the restoration of GSH in hepsin^−/−^ mice was less effective than in wild-type mice at 4 and 6 h following APAP treatment (Fig. 1F). In line with these observations, the nitrotyrosine level in the liver of hepsin^−/−^ mice was approximately 2- to threefold higher than that measured for wild-type mice, with this difference becoming noticeable as early as 2 h post-APAP administration (Fig. 1G). Taken together, these findings indicated that hepsin^−/−^ mice experience heightened oxidative stress in the early stages of APAP exposure, suggesting that endogenous hepsin might protect against toxicity during the early stages of APAP exposure, thereby significantly influencing survival outcomes in drug-induced toxicity cases.

### Administering hepsin mitigates drug-induced liver damage by acetaminophen in hepsin^−/−^ mice.

**Fig. 2:**
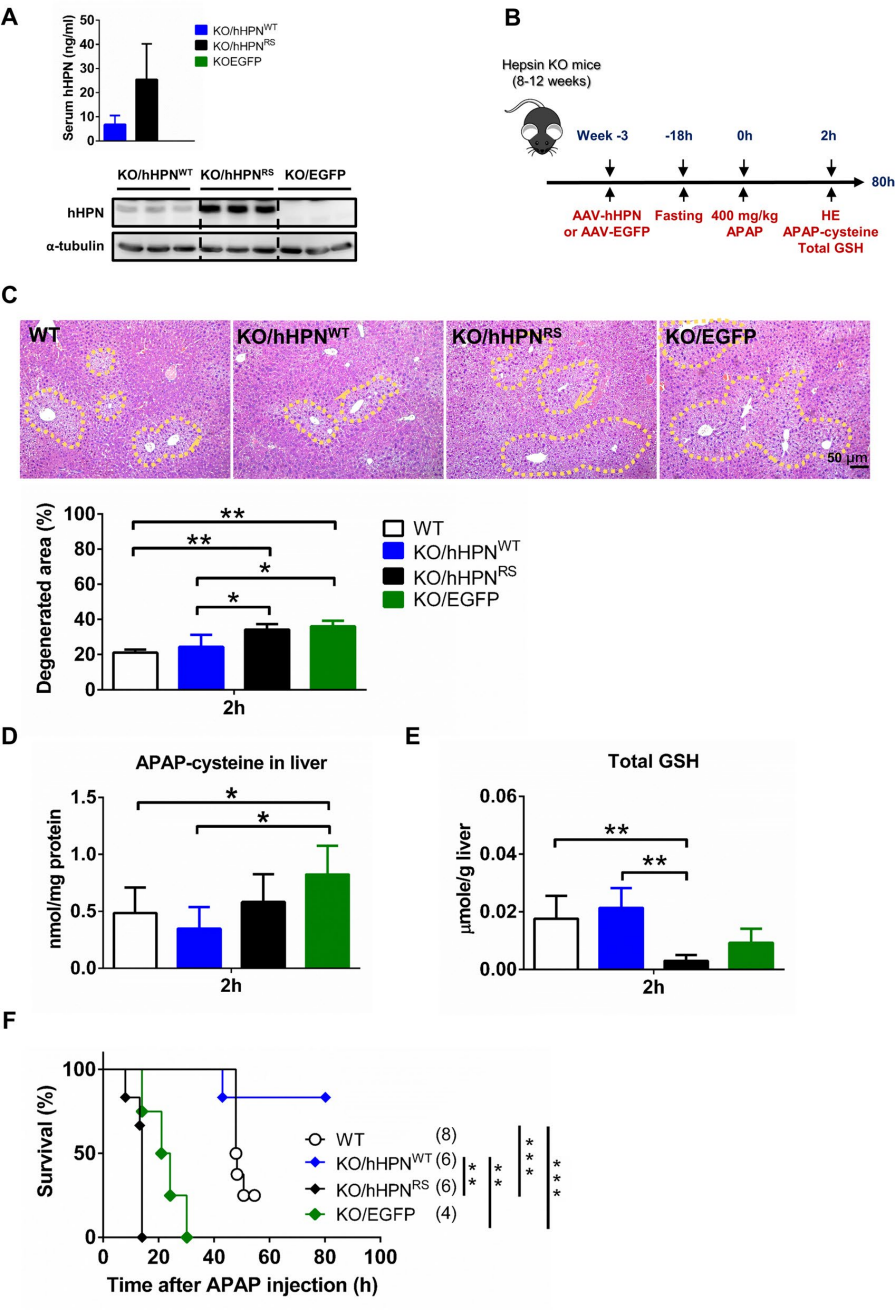
AAV-mediated liver-specific administration of hepsin to hepsin^−/−^ mice decreases their susceptibility to APAP, the area of liver degeneration, APAP-cysteine formation, and total glutathione in mouse liver**.** Hepsin^−/−^ mice were administered AAV-hHPN^WT^, AAV-hHPN^RS^ or AAV-EGFP for 3 weeks, followed by 400 mg/kg APAP treatment. (**A**) Human hepsin (hHPN) levels in serum and liver lysate prior to administering APAP (n = 3–5 per group). (**B**) Experimental timeline. (**C**) Images of liver pathology in sections assessed by hematoxylin and eosin staining and quantification as a percentage of the degenerated area at 2 h after 400 mg/kg APAP treatment. (**D**) Amounts of APAP-cysteine at 2 h after 400 mg/kg APAP treatment (n = 4–5 per group). (**E**) Total glutathione (GSH) at 2 h after 400 mg/kg APAP treatment (n = 4–5 per group). (**F**) Survival rate after 400 mg/kg APAP treatment. In the survival analysis, sample sizes for each group are indicated in brackets, and statistical significance was determined using the log-rank test, with significance levels represented by asterisks: **p < 0.01, ***p < 0.001. Data are presented as the mean ± SD in bar charts, with significance levels denoted by asterisks: *p < 0.05, **p < 0.01

We employed an adeno-associated virus vector (AAV2/8) to deliver and express either human wild-type hepsin (hHPN^WT^; AAV-hHPN^WT^) or a loss-of-function mutant (hHPN^RS^; AAV-hHPN^RS^). The hHPN^RS^ mutant was engineered to produce hepsin without functional activity by introducing two specific mutations in critical regions of its proteolytic activity. We validated the deficient cleavage process and the loss of protease function in this mutant form of HPN in our previous publication (Hsu et al. 2012), where it was used as a control in our experiments. An AAV encoding enhanced green fluorescent protein (EGFP; AAV-EGFP) was used as the vector control. The presence of human hepsin in hepsin^−/−^ mouse serum and liver lysate was confirmed and quantified via enzyme-linked immunosorbent assay (ELISA) and western blot on Day 14 after administering the same dose of AAV-hHPN^WT^, AAV-hHPN^RS^ or AAV-EGFP (Fig. 2A). These results confirmed that our AAV-hHPN vector design successfully enabled the overexpression of human hepsin in mouse liver. As expected, we observed that the loss-of-function mutant form of hHPN^RS^ exhibited higher protein levels compared to the wild-type hHPN^WT^, both in serum and liver lysate. This difference may primarily be due to the auto-activation and subsequent degradation of hHPN^WT^ (Vu et al. 1997; Wang et al. 2019), and it also provides evidence for the deficiency of protease activity in the loss-of-function mutant form of hHPN^RS^.

Following the experimental setup, each group of hepsin^−/−^ mice were administered APAP at 400 mg/kg, after which the survival rates were closely monitored (Fig. 2B). Overexpression of hHPN^WT^ in hepsin^−/−^ mice significantly increased their tolerance to APAP, as evidenced by a marked reduction in liver degeneration area and APAP-cysteine adduct levels at 2 h post-APAP injection compared to the two control groups (Fig. 2C, D). Furthermore, wild-type hepsin expression appeared to mitigate the depletion of GSH, a protective effect not observed in hepsin^−/−^ mice expressing hHPN^RS^ or in the vector-control group (Fig. 2E). Consistently, overexpression of hHPN^WT^ in hepsin^−/−^ mice resulted in a significant increase in APAP tolerance, with survival rates exceeding 80% even after 80 h (Fig. 2F). In contrast, survival rates were similar between vector-control mice and those expressing the loss-of-function mutated hepsin, with all mice in these groups succumbing within 30 h (Fig. 2F). This clearly indicated that the expression of hHPN^WT^ could notably reduce the early symptoms of APAP-induced hepatotoxicity in hepsin^−/−^ mice, i.e., it effectively diminished the severity of these symptoms to levels comparable to those observed in wild-type mice. These results established a crucial connection between the serine protease function of hepsin and protection against APAP-induced hepatotoxicity.

### Transcriptome analysis of hepsin^−/−^ and wild-type mice during the early period after APAP exposure

**Fig. 3:**
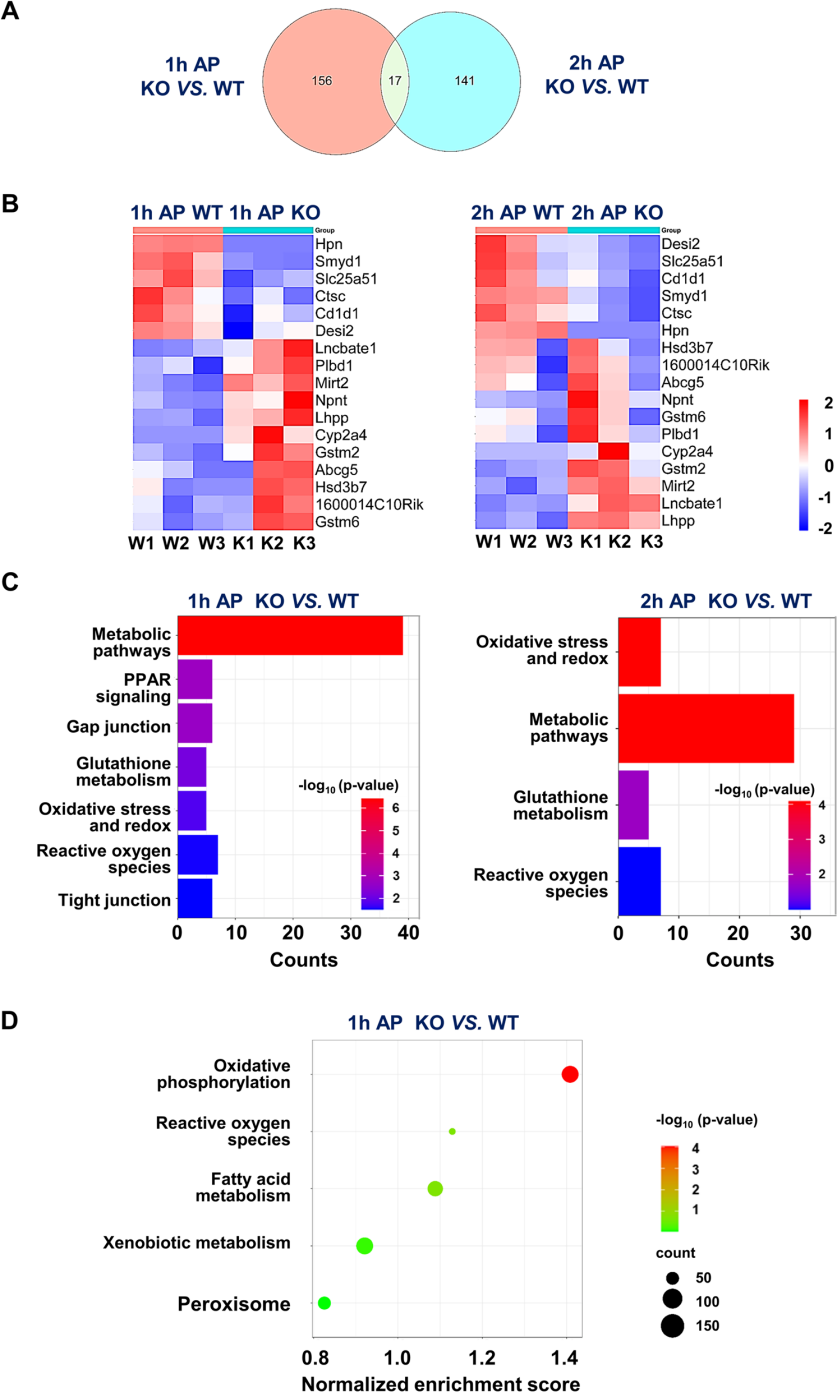
Liver transcriptomes after 1 and 2 h after 400 mg/kg APAP treatment**.** (**A**) Comparison between transcriptomes of hepsin^−/−^ and wild-type mice. The Venn diagram shows the differential expression of transcripts between these two mouse groups assessed at 1 and 2 h after 400 mg/kg APAP treatment. The overlap of the differentially expressed transcripts is shown. (**B**) Unsupervised clustering analysis of the 17 differentially expressed transcripts that were common between the 1- and 2- hour time points after 400 mg/kg APAP treatment. (**C**) Functional enrichment analysis of differentially expressed transcripts at 1 and 2 h after 400 mg/kg APAP treatment. (**D**) Results from a gene set enrichment analysis for hepsin^−/−^ and wild-type mice at 1 h after 400 mg/kg APAP treatment. APAP is indicated as AP. Each group consisted of three mice

To clarify the function of hepsin in the early stages of APAP-induced hepatotoxicity, a transcriptome analysis employing RNA sequencing was conducted within the first 2 h post-APAP administration. Our results demonstrated a significant downregulation of the PI3K/AKT pathway (*p* = 0.049) at one hour, and of mTOR (*p* = 0.033 at one hour and *p* = 0.021 at two hours) following APAP treatment in wild-type mice (Fig. S4). These findings align with previous reports indicating that downstream pathways, particularly PI3K/AKT and mTOR, are responsive to hepsin downregulation (Fig. S2) (Li et al. 2020b). Furthermore, the analysis indicated that, at 1 and 2 h following APAP administration, hepsin^−/−^ mice displayed 156 and 141 differentially expressed genes, respectively, when compared with wild-type mice at the same time points. Notably, 17 of these genes were differentially expressed at both 1 and 2 h, underscoring their significance in the early stage after APAP administration (Fig. 3A). These 17 genes were further subjected to Gene Ontology analysis, and temporal data are depicted in a heat map. This analysis revealed significant differences in the liver transcriptomes of wild-type and hepsin^−/−^ mice post-APAP exposure. Particularly, genes involved in lipid metabolism (*Abcg5*), drug metabolism (*Cyp2a4*), GSH metabolism (*Gstm2* and *Gstm6*), and oxidative phosphorylation (*Lhpp*) were expressed at higher levels in the liver of hepsin^−/−^ mice compared with wild-type mice. These results further suggest that hepsin^−/−^ mice experienced increased oxidative stress in the early hours following APAP administration, leading to more severe drug-induced liver damage (Fig. 3B).

Next, a functional enrichment analysis was conducted on the genes that were differentially expressed at 1 and 2 h post-APAP administration in hepsin^−/−^ mice. The analysis of data for both time points revealed significant upregulation of genes in various pathways, especially those related to general metabolism, GSH metabolism, and oxidative stress, such as oxidative phosphorylation and ROS, in hepsin^−/−^ mice compared with wild-type mice (Fig. 3C). Gene set enrichment analysis highlighted the early activation of oxidative stress-related pathways, including oxidative phosphorylation and ROS generation, as early as 1-h post-APAP exposure. This upregulation aligned with Gene Ontology analysis data and our findings on the early stages of APAP-induced hepatotoxicity in hepsin^−/−^ mice (Fig. 3D).

Notably, the results from our functional enrichment analysis highlighted significant distinctions in the GJ pathway transcriptome between hepsin^−/−^ and wild-type mice at 1-h post-APAP administration (Fig. 3C, Fig. S5). This phenomenon was especially noteworthy given the body of research linking the expression of Cx32, which is an essential structural protein in hepatocyte GJs, with the underlying mechanisms of liver toxicity induced by APAP. Previous research has suggested that hepatic GJ proteins might facilitate the spread of free radicals, thereby exacerbating the severity of drug-induced liver damage (Patel et al. 2012). The notable increase in Cx32 level in hepatocytes of hepsin^−/−^ mice, along with our previous work establishing hepsin as a regulator of the cellular abundance of hepatic GJ proteins (Hsu et al. 2012), led us to propose that hepsin could significantly diminish the severity of APAP-induced hepatotoxicity by regulating the abundance of hepatic GJ proteins, which may alter the distribution of free radicals between neighboring hepatocytes, thereby potentially increasing oxidative stress.

### Inhibiting the elevated expression of GJ components in hepsin^−/−^ mice reduces APAP toxicity by limiting oxidative stress in the liver

**Fig. 4:**
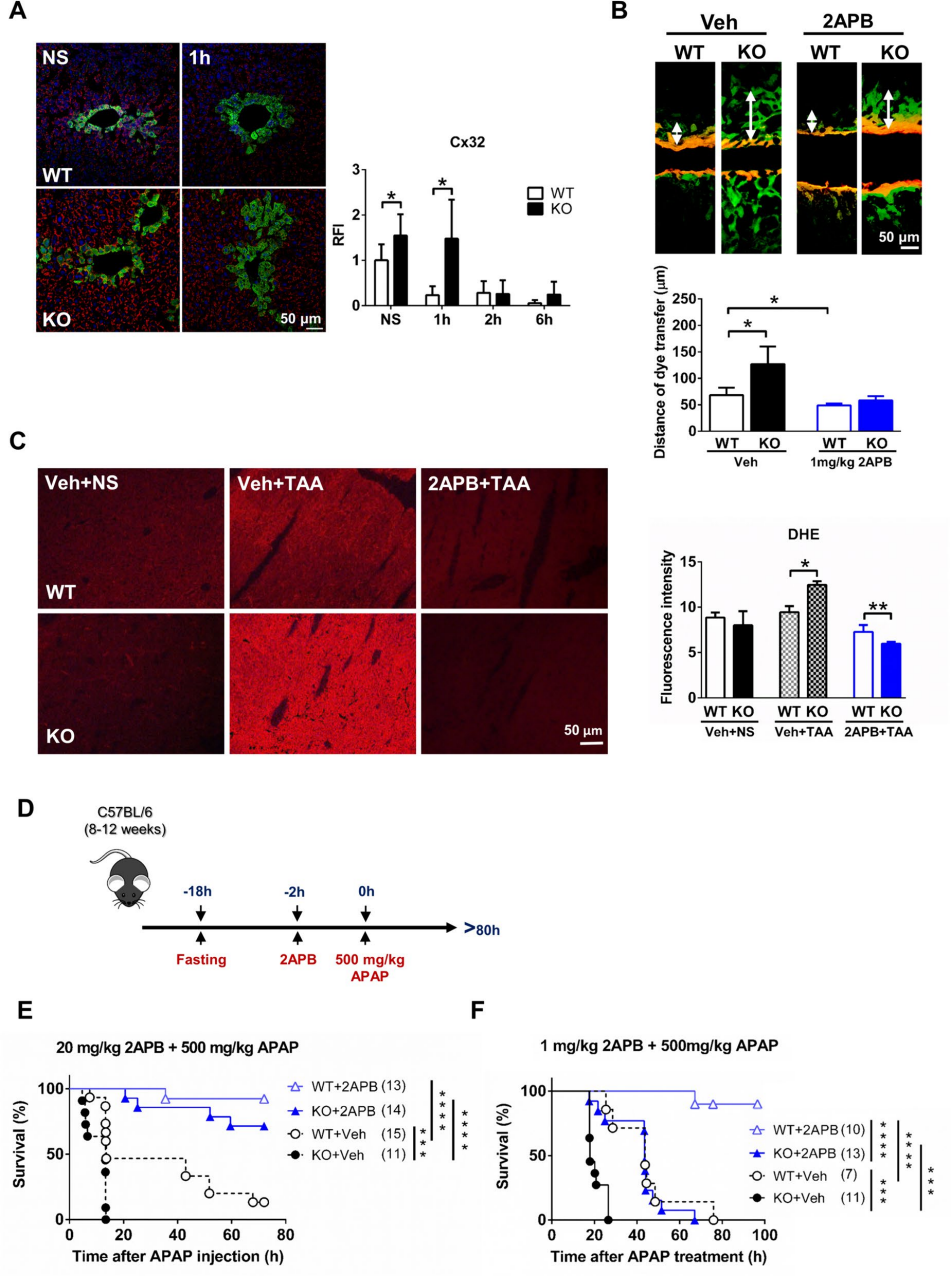
Hepsin^−/−^ mice exhibit delayed downregulation of connexin 32 (Cx32) after injection with APAP, and blockade of GJ intercellular communication formed by Cx32 alleviates liver injury and decreases the diffusion of reactive oxygen species**.** (**A**) Representative immunofluorescence photomicrographs of mouse liver sections stained for Cx32, quantified as relative fluorescence intensity (RFI) at the indicated time points after 400 mg/kg APAP treatment. NS, normal saline control group (n = 4–8 per group). (**B**) An incision loading/dye transfer test was conducted to evaluate functional GJ intercellular communication in liver tissue 3 h after injection with 1 mg/kg 2APB. Representative immunofluorescence photomicrographs were used to quantify the permeability of Lucifer yellow relative to rhodamine-dextran, representing GJ connectivity between hepatocytes. Veh, vehicle (0.1% DMSO) (n = 3–4 per group). (**C**) Representative immunofluorescence photomicrographs of mouse liver sections stained with dihydroethidium (DHE) show the amount of ROS 6 h after injection of mice with 500 mg/kg thioacetamide (TAA), along with 1 mg/kg 2APB or vehicle control (Veh) (n = 4–8 per group). (**D**) Experimental timeline. (**E, F**) Survival rate of wild-type and hepsin^−/−^ mice treated with 20 mg/kg or 1 mg/kg 2APB, followed by administration with 500 mg/kg APAP. In the survival analysis, sample sizes for each group are indicated in brackets, and statistical significance was determined using the log-rank test, with significance levels represented by asterisks: ***p < 0.001, ****p < 0.0001. Data are presented as the mean ± SD in bar charts, with significance levels denoted by asterisks: *p < 0.05, **p < 0.01

To investigate GJ expression variations after APAP administration in wild-type and hepsin^−/−^ mice, immunofluorescence staining for Cx32 was assessed at different time points. In wild-type mice, Cx32 abundance in hepatocytes decreased substantially as early as 1-h post-APAP treatment, reaching a 20% reduction compared to the steady-state or control group. This decrease in GJ expression was consistently evident at later time points, 2 to 6 h post-APAP exposure (Fig. 4A, Fig. S6). Considering that hepatocyte GJs aid the transmission of oxidative stress molecules in mice (Igarashi et al. 2014; Patel et al. 2012), our observation that APAP decreased GJ abundance suggested that hepatocytes mount a defense against the spread of oxidative stress molecules within liver tissue during the early stages of APAP exposure by reducing the expression of Cx32 (and consequently GJs).

Compared with the natural protective mechanism of wild-type mice, hepsin^−/−^ mice had a different response to Cx32 expression after APAP exposure. In the normal saline control group, hepsin^−/−^ mice showed a 1.5-fold increase in hepatocyte Cx32 expression relative to the wild-type control group. After APAP administration, however, the decrease in GJ expression in hepatocytes of hepsin^−/−^ mice was more gradual compared with that in wild-type mice. Consequently, there was an approximate threefold increase in Cx32 expression in wild-type mice observed at 1-h post-APAP treatment. In contrast, a significant reduction in Cx32 expression in hepsin^−/−^ mice was not evident until 2 h post-APAP administration (Fig. 4A, Fig. S6). Therefore, we hypothesized that the excessive expression of GJs post-APAP exposure in hepsin^−/−^ mice could be a key factor contributing to the increased severity of liver damage observed following APAP administration.

To further substantiate that inhibiting GJs is an effective means of protecting against APAP-induced hepatotoxicity, we utilized the GJ functional inhibitor 2-aminoethoxydipenyl borate (2APB) (Leytus et al. 1988). To confirm the inhibitory effects of 2APB, an incision loading/dye transfer experiment was conducted. Gap junctional transmission was substantially greater in hepsin^−/−^ mice, likely due to the elevated expression of Cx32; however, a 3-h treatment with 2APB led to a substantial decrease in GJ transmission in both wild-type and hepsin^−/−^ mice (Fig. 4B). Given the characteristics of short-lived free radical species, which are challenging to confirm as transferring between cells through gap junctions after APAP overdose, we employed a thioacetamide-induced hepatotoxicity model. This model, known for producing a higher amount of ROS, was used to assess oxidative stress in mouse liver using the ROS probe dihydroethidium, as demonstrated in previous studies (Patel et al. 2012)**.** A 6-h treatment with thioacetamide led to a significant increase in ROS levels compared with the control group, but pre-treatment with 2APB effectively mitigated this oxidative stress in the liver of both wild-type and hepsin^−/−^ mice. These results indicated that the administration of 2APB effectively inhibited the transmission of oxidative stress molecules by hepatocyte GJs and impacted oxidative stress in the liver. Notably, the hepsin^−/−^ mice exhibited a more pronounced increase in ROS levels after thioacetamide treatment compared with wild-type mice, which aligned with our hypothesis that hepsin^−/−^ mice experience heightened ROS stress in the early stages of APAP exposure due to their elevated expression of GJ proteins (Fig. 4C).

A subsequent experiment was conducted to explore the correlation between the observed overexpression of GJs and the ensuing heightened liver damage in hepsin^−/−^ mice following APAP administration. Both wild-type and hepsin^−/−^ mice received an intraperitoneal injection of 2APB (20 mg/kg), followed by a dose of APAP (500 mg/kg) 2 h later (Fig. 4D). For both the wild-type and hepsin^−/−^ mice treated with 2APB, the survival rate increased significantly compared with the vehicle control group at 72 h post-APAP administration (Fig. 4E). This indicated that the functional inhibition of GJ proteins not only protects against APAP-induced hepatotoxicity but also effectively mitigates the increased sensitivity to APAP as seen in hepsin^−/−^ mice. However, when administering a lower dose of 2APB, i.e., 1 mg/kg, the protective effect on APAP tolerance was observed only in wild-type mice, for which survival rate was 100% at 60 h post-2APB administration; in contrast, the survival rate was only 8% for hepsin^−/−^ mice. At 80 h post-administration, the survival rate was 90% for wild-type mice but dropped to 0% for hepsin^**−/−**^ mice (Fig. 4F). This difference was likely attributable to the insufficient inhibition of excessive GJ proteins in hepsin^−/−^ mice in response to the lower 2APB dose of 1 mg/kg, lending further support to the hypothesis that hepsin may help reduce APAP toxicity by regulating the expression of hepatic GJ proteins. Collectively, these results affirmed that hepsin plays a crucial role in regulating GJ communication, which in turn impacts oxidative stress. This regulation by hepsin contributed to early protection against APAP-induced liver damage.

### Administering hepsin to wild-type mice increases APAP tolerance and downregulates GJ expression

**Fig. 5:**
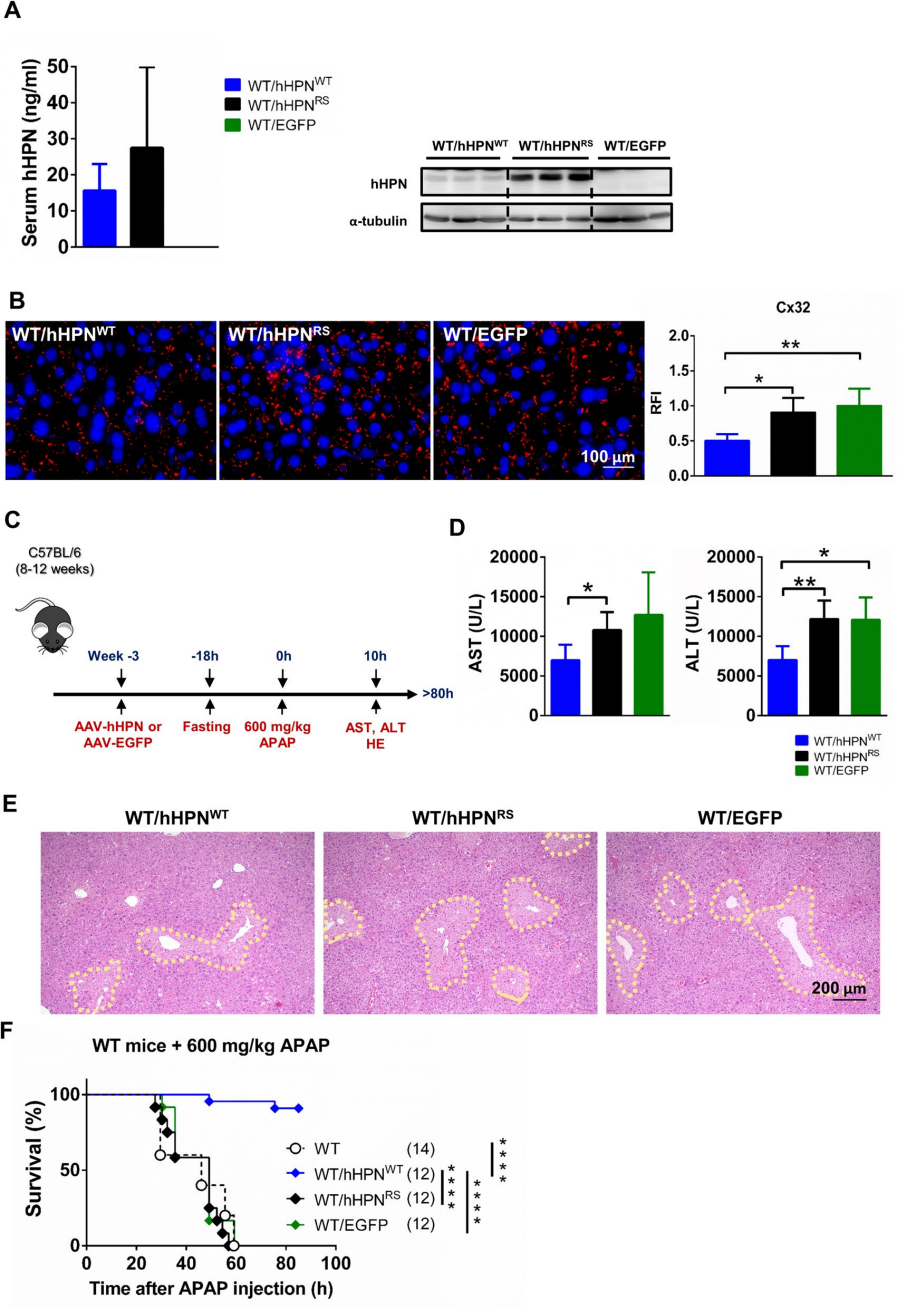
AAV-mediated liver-specific administration of hepsin to adult wild-type mice alleviates APAP-induced liver injury**.** Adult wild-type mice were administered AAV-hHPN^WT^, AAV-hHPN^RS^ or AAV-EGFP for 3 weeks and then treated with 600 mg/kg APAP. (**A**) Human hepsin (hHPN) levels in serum and liver lysate prior to administering APAP (n > 15 per group for serum level detection and n = 3 per group for liver lysate detection). (**B**) Representative immunofluorescence photomicrographs of mouse liver sections stained for Cx32 after liver-specific administration of hepsin by AAV. Quantification is represented by relative fluorescence intensity (RFI) (n = 4–7 per group). (**C**) Experimental timeline. (**D**) Measurement of serum AST and ALT levels at 10 h after injection with 600 mg/kg APAP (n = 5 per group). (**E**) Images of liver pathology in sections assessed by hematoxylin and eosin staining at 10 h after 600 mg/kg APAP treatment, show the degenerated area indicated by a yellow dashed line. (**F**) Survival rate. In the survival analysis, sample sizes for each group are indicated in brackets, and statistical significance was determined using the log-rank test, with significance levels represented by asterisks: ****p < 0.0001. Data are presented as the mean ± SD in bar charts, with significance levels denoted by asterisks: *p < 0.05, **p < 0.01

Considering the potential clinical and medical applications, we evaluated a therapeutic strategy involving the administration of hepsin to wild-type adult mice and assessed its potential to mitigate APAP-induced liver injury. Three weeks after transduction with AAV, the serum levels and liver lysate of human hepsin were confirmed and quantified via ELISA and western blot following the administration of the same dose of AAV-hHPN^WT^, AAV-hHPN^RS^ or AAV-EGFP (Fig. 5A). Additionally, in wild-type mice that overexpressed hHPN^WT^, there was a significant reduction in Cx32 expression, i.e., approximately 50 ± 9% compared with both the AAV-hHPN^RS^ and the vector-control (AAV-EGFP) groups (Fig. 5B). These findings suggest that administering hepsin can effectively regulate GJ expression in wild-type mice, highlighting its potential as a therapeutic target to protect against APAP-induced hepatotoxicity, potentially in human patients.

To assess the ability of hepsin overexpression to protect against APAP-induced hepatotoxicity, a lethal dose of 600 mg/kg APAP was first administered to mice in each experimental group (Fig. 5C). Serum aspartate aminotransferase and alanine transaminase levels were measured at 10 h post-APAP administration. The group of wild-type mice expressing hHPN^WT^ exhibited significantly lower levels of both enzymes, which reflected reduced liver damage, compared to the two control groups (Fig. 5D). Consistently, there was a marked reduction in liver degeneration area at the same time point in wild-type mice expressing hHPN^WT^, compared to the two control groups (Fig. 5E). Additionally, a survival-rate analysis revealed a significant increase in APAP tolerance in mice expressing hHPN^WT^, with more than 80% surviving at the 80-h post-administration time point. In contrast, the survival of mice expressing a hHPN^RS^ was similar to that of the vector-control group (AAV-EGFP), with all mice succumbing within approximately 60 h (Fig. 5F). These results demonstrated that administering hepsin afforded protection against APAP-induced liver damage in adult wild-type mice and ultimately prolonged their survival. This protective effect is achieved through the downregulation of GJ expression, suggesting that hepsin could be a viable therapeutic agent for APAP-induced hepatotoxicity.

### Novel combination therapy with hepsin and low doses of NAC improves therapeutic effectiveness and extends survival in APAP-induced lethality

**Fig. 6:**
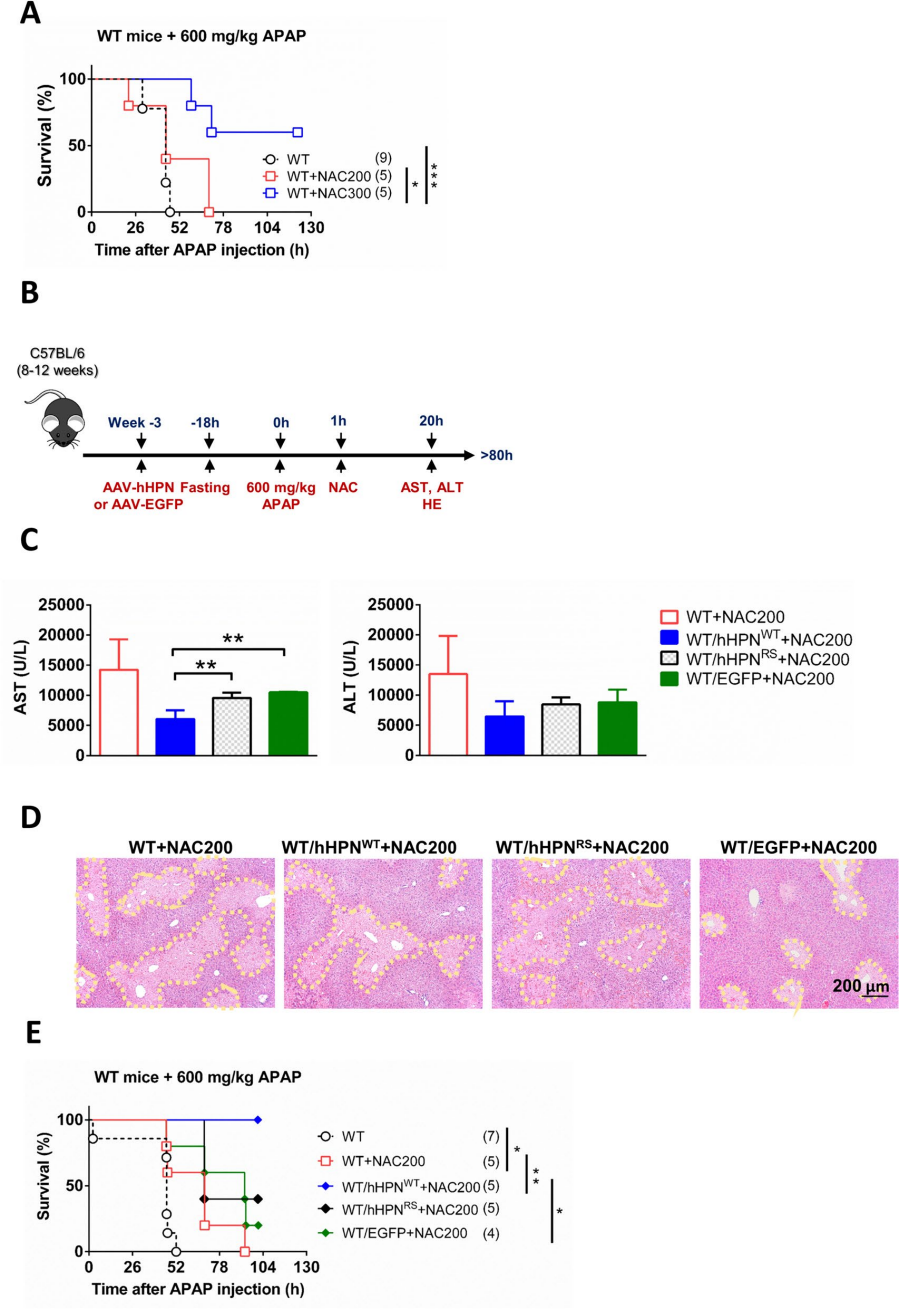
Combination of AAV-mediated liver-specific administration of hepsin and post-injection with N-acetyl-cysteine has a superior therapeutic effect compared with N-acetyl-cysteine treatment alone**.** (**A**) The survival rate after 600 mg/kg APAP administration, followed by different doses of NAC therapy provided 1 h post-APAP treatment. (**B**) Experimental timeline. Adult wild-type mice were administered AAV-hHPN^WT^, AAV-hHPN^RS^, or AAV-EGFP for 3 weeks, followed by 600 mg/kg APAP treatment for 1 h, after which the mice were treated with NAC. (**C**) Measurement of serum AST and ALT at 20 h after APAP treatment (n = 3–5 per group). (**D**) Images of liver pathology in sections assessed by hematoxylin and eosin staining at 20 h after APAP treatment. (**E**) Survival rate. In the survival analysis, sample sizes for each group are indicated in brackets, and statistical significance was determined using the log-rank test, with significance levels represented by asterisks: *p < *0.05,* **p < 0.01, ***p < *0.01*. Data are presented as the mean ± SD in bar charts, with significance levels denoted by asterisks: **p < 0.01

The standard clinical approach for treating APAP-induced liver injury involves administering NAC. Given that the effectiveness of NAC for treating APAP-induced liver injury is confined to modest improvements within a brief period after onset and considering that high doses of NAC can potentially negatively impact hepatocyte metabolism and liver regeneration (Jaeschke et al. 2020), we pursued a combination therapy strategy involving the administration of hepsin, aiming to amplify the therapeutic efficacy while employing lower doses of NAC. We first established a therapy model of NAC in our animal model. In an experimental model using a lethal dose of 600 mg/kg APAP in adult wild-type mice, administering a high dose of 300 mg/kg NAC at 1-h post-APAP exposure resulted in significant therapeutic effectiveness, with 100% survival. In contrast, mice in the control group died within 50 h. Moreover, this high dose of NAC continued to show therapeutic benefits for nearly 130 h post-APAP exposure, with a survival rate of 50–60%. In contrast, administering a lower dose of 200 mg/kg NAC was insufficient to prevent mortality (Fig. 6A).

To assess the therapeutic effectiveness of combining hepsin with a reduced dose of 200 mg/kg NAC (Fig. 6B), we measured serum AST and ALT levels in the early stage post-APAP administration to evaluate protection against 600 mg/kg APAP-induced hepatotoxicity. The group of wild-type mice treated with hepsin and a reduced dose of 200 mg/kg NAC exhibited significantly lower levels of both enzymes, indicating reduced liver damage compared to the other groups (Fig. 6C). This finding was consistent with the marked reduction in liver degeneration observed in the combination therapy group at the same time point (Fig. 6D). Additionally, survival-rate analysis revealed a notable increase in therapeutic effectiveness, with 100% survival for wild-type mice over a 100-h period following a lethal dose of APAP when treated with the combination of hepsin and 200 mg/kg NAC. This level of improvement was not observed in the combination therapy groups administered with AAV-hHPN^RS^ or the vector control (AAV-EGFP) (Fig. 6E). Thus, the combined administration of hepsin with NAC optimized the therapeutic outcome, enhancing effectiveness beyond what was achievable with NAC alone. This result highlights the potential of combined therapy with hepsin and NAC to treat APAP-induced liver injury, paving the way for promising future research.

## Discussion

Here, we present the first evidence of the physiological role of endogenous hepsin in redox homeostasis and its potential protective effect early after APAP exposure, which is crucial for mitigating liver injury. Our research uniquely highlights hepsin's role in counteracting APAP-induced liver damage, introducing an innovative combination therapy with NAC that enhances overall therapeutic effectiveness, marking a significant advancement in therapeutic approaches. Studies with mouse models have shown that hepsin is important for maintaining hepatic structural homeostasis, liver metabolism, and adipocyte browning (Hsu et al. 2012; Li et al. 2020a), although its precise physiological role is not fully understood. Considering that hepsin^−/−^ mice have been reported to have dramatically reduced hepatic glycogen stores (Li et al. 2020b), and that further glycogen depletion after overnight fasting may contribute to more severe GSH depletion (Hinson et al. 1983), we conducted survival assays using two different APAP treatment doses in non-fasting wild-type and hepsin^−/−^ mice (Fig. S7) to clarify the relationship between reduced glycogen, GSH depletion, and increased susceptibility to APAP in hepsin^−/−^ mice. Despite the absence of fasting, APAP hepatotoxicity remained more severe in the non-fasting hepsin^−/−^ mice (Fig. S7). Furthermore, transcriptome analysis revealed no significant differences in the expression levels of genes related to GSH synthetic pathways between wild-type and hepsin^−/−^ mice (Fig. S8).

Our previous research showed that hepsin^−/−^ mice exhibited decreased HGF-cMet signaling, with reduced levels of activated HGF and phosphorylated cMet, resulting in a two-fold increase in GJ expression compared to wild-type mice (Hsu et al. 2012). HGF/c-Met signaling is crucial for regulating cellular redox homeostasis and oxidative stress. Extensive research has indicated that the HGF-cMet pathway protects against oxidative stress by upregulating antioxidant proteins like superoxide dismutase and catalase and stimulating GSH biosynthesis (Gomez-Quiroz et al. 2008; Valdés-Arzate et al. 2009). Additionally, HGF/c-Met signaling regulates the NADPH oxidase system, initiating an Nrf2-mediated protective response. This regulation is absent in c-Met-deficient primary mouse hepatocytes, leading to the overproduction of ROS, increased oxidative stress, and heightened sensitivity to apoptosis-promoting agents. Thus, this regulation by the HGF/c-Met pathway constitutes a protective mechanism in normal cells (Clavijo-Cornejo et al. 2013). Supporting this, data from our functional enrichment analysis of the transcriptome revealed notable upregulation of oxidative phosphorylation and the oxidative damage response in hepsin^−/−^ mice treated with saline (data not shown). As expected, hepsin^−/−^ mice displayed heightened oxidative stress after APAP exposure compared to wild-type mice, indicated by a significant decrease in GSH and an increase in nitrotyrosine levels, leading to more severe liver damage and mortality.

Our functional enrichment and gene set enrichment analyses revealed differences in metabolic pathways, particularly fatty acid metabolism, between hepsin^−/−^ and wild-type mice following APAP exposure. Previous studies have demonstrated that APAP intoxication disrupts the temporal dynamics of hepatic lipid metabolism (Xiong et al. 2014), affecting both the balance of hepatic free fatty acids and the expression of key lipid metabolism genes in the liver (Suciu et al. 2015). Moreover, APAP-induced mitochondrial damage has been shown to partly result from the suppression of PPARα-regulated pathways, leading to irreversible inhibition of fatty acid oxidation. The protective effect of PPARα agonists against APAP-induced toxicity emphasizes the importance of fatty acid metabolism in this process (Patterson et al. 2012). Interestingly, we noted upregulation of PPAR signaling pathways in hepsin^−/−^ mice just 1-h post-APAP exposure (Fig. 3C), suggesting a possible compensatory mechanism or different metabolic response due to the absence of hepsin, potentially influencing vulnerability to and progression of APAP-induced liver injury.

Given that hepsin^−/−^ mice were reported to have elevated mRNA levels of mitochondrial *Cpt1b*, *Cpt2*, and *Cox7a1* in adipose tissues compared with wild-type mice, suggesting a regulatory role for hepsin in metabolism (Li et al. 2020b), we conducted a comparative analysis of the liver transcriptome to clearly characterize mitochondrial modulation in our study. We focused on mitochondrial genes between wild-type and hepsin^−/−^ mice under normal saline conditions and at 1 and 2 h post-APAP treatment (Figure S9). Our analysis showed no significant differences in the expression of mitochondrial genes or related pathways between wild-type and hepsin^−/−^ mice at any time point, as determined by transcriptome analysis, pathway enrichment, and GSEA.

While our study highlights the significance of hepsin in the context of liver protection and its potential therapeutic implications in APAP-induced hepatotoxicity, there are limitations to consider when evaluating hepsin as a candidate drug or therapeutic strategy. As a serine protease, hepsin presents challenges for administration as an intact or activated protein due to its tendency to auto-activate, degrade, and its overall instability (Li et al. 2021; Wang et al. 2019). This necessitates the development of small molecule inhibitors or mRNA delivery strategies to effectively harness its potential. Furthermore, as a member of the TMPRSS family of serine proteases, hepsin plays crucial roles in various biological processes, including maintaining liver architecture, regulating cell growth, and influencing other serine proteases and lipid metabolism (Hsu et al. 2012; Li et al. 2021; Torres-Rosado et al. 1993). Despite its promise, further research is needed to fully understand hepsin's mechanisms of action and safety profile.

Gap junctions and their constituent molecular channels, which are primarily composed of Cx, are crucial for tissue function and have been studied as drug targets for decades (Cooreman et al. 2019; Van Campenhout et al. 2021). In our study, we observed that Cx32 levels decreased within 1 h in APAP-treated wild-type mice, whereas in hepsin-/- mice, this reduction occurred at 2 h post-treatment (Fig. 4A). The degradation of Cx32 from the plasma membrane is a key process in this rapid turnover, which is crucial for the regulation of GJIC. This allows cells to quickly adapt to changing physiological conditions by altering the composition and function of gap junctions (Fallon and Goodenough 1981). Furthermore, disruption of adherens junctions, including E-cadherin and α-catenin, rapidly decreases gap junction plaques, leading to reduced Cx32 levels. (Fujimoto et al. 1997). Several studies have suggested the involvement of connexin and hemichannel signaling in APAP-induced acute liver failure. Connexins enable direct intercellular communication of metabolites, nucleotides, nutrients, and secondary messengers, regulating apoptosis, inflammation, and cellular growth, while also amplifying liver inflammation and cell death (Maes and Vinken 2017; Patel et al. 2012). Hemichannels also contribute to liver damage by facilitating the influx of toxic substances and the loss of essential metabolites. ATP release through connexin hemichannels depletes intracellular ATP, leading to necrosis and inflammation (Kalvelyte et al. 2003; Maes et al. 2017). In contrast, Cx32 has been reported the protective effects against APAP-induced liver toxicity by transporting GSH between hepatocytes, although the outcomes have been somewhat contradictory (Igarashi et al. 2014). The increased expression of Cx32 in hepsin^−/−^ mice seemed to exacerbate liver injury rather than having a cytoprotective effect in the context of APAP-induced liver injury. Our research showed that 2APB, a small-molecule inhibitor of GJ intercellular communication, effectively halted oxidative stress transmission and improved survival in both hepsin^−/−^ and wild-type mice. However, lower doses of 2APB were ineffective in normalizing elevated Cx32 levels in hepsin^−/−^ mice, indicating limited therapeutic efficacy. These results highlight hepsin's crucial role in controlling APAP-induced liver toxicity by regulating the cellular abundance of GJ proteins, which can affect the transmission of toxic metabolites or cell death signals and potentially increase oxidative stress.

Recent reports indicate that ROS produced during liver transplantation were transferred to neighboring cells through Cx32 channels, aggravating oxidative stress and inflammation (Huang et al. 2023). This aligns with previous findings that gap junction inhibition protected against postoperative acute kidney injury by reducing ROS transmission between cells (Yuan et al. 2019). Our study revealed pronounced oxidative stress in mice exposed to APAP, which contrasts with previous research showing that mimic peptides targeting necrotic cells in APAP-induced hepatotoxicity do not significantly alter liver oxidative status or GSH level (Maes et al. 2017). This discrepancy suggests that factors beyond connexins may contribute to hepsin's protective role. Additionally, we extended our focus to RIP1 and RIP3, which are key inducers of necroptosis in the early phase of APAP toxicity, manifesting effects within 2 h of administration (Liu et al. 2019). However, our observations revealed no substantial differences in RIP1 and RIP3 levels, indicating hepsin's role may not involve necroptosis modulation (Fig. S10). Histological assays showed a lack of severe immune-cell infiltration in the liver of hepsin^–/–^ mice compared to wild-type mice within 6 h post-APAP exposure, suggesting the need for further investigation of inflammation in hepsin-mediated APAP intoxication.

## Conclusions

**Fig. 7:**
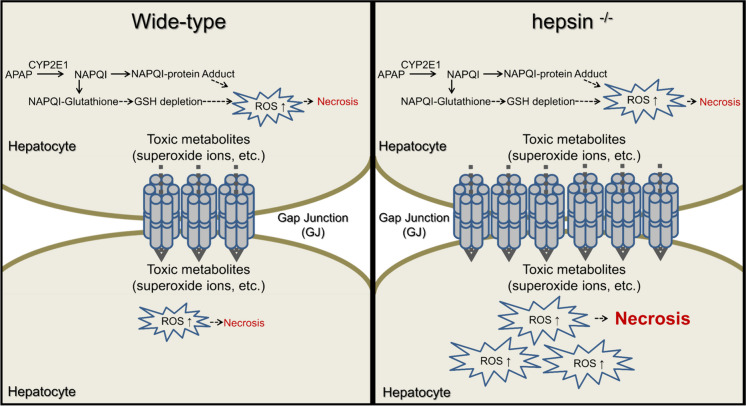
The diagram illustrates the differing severity of liver damage in wild-type versus hepsin^−/−^ mice after APAP exposure, emphasizing the role of gap junctions in exacerbating liver damage. Our results suggest a crucial role for hepsin in modulating the abundance of hepatic gap junctions and reducing oxidative stress, thereby offering early protection against acetaminophen-induced hepatotoxicity

Our study reveals the critical role of hepsin, a type II transmembrane serine protease, in protecting against APAP-induced liver injury by decreasing cellular GJ abundance in response to reactive oxygen stress in the liver. The combination of hepsin with NAC shows a synergistic effect, providing more effective protection than NAC alone, thus offering new avenues for enhancing current treatment approaches. Although this novel approach will require further validation and exploration in clinical trials, it holds promise for improving the management of APAP-induced liver injury (Fig. 7).

## Supplementary Information

Below is the link to the electronic supplementary material.

Supplementary file1 (PDF 16388 KB)

## Data Availability

Data will be made available on request.

## Abbreviations

*APAP*: Acetaminophen
*GJ*: Gap-junction
*NAC*: N-acetylcysteine
*GSH*: Glutathione
*NAPQI*: N-acetyl-p-benzoquinone imine
*ROS*: Reactive oxygen species
*Nrf2*: Nuclear factor E2-related factor 2
*Cx32*: Connexin 32
*TTSP*: Type II transmembrane serine protease
*HGF*: Hepatocyte growth factor
