# Supplementary material for: Hepsin as a potential therapeutic target for alleviating acetaminophen-induced hepatotoxicity via gap-junction regulation and oxidative stress modulation
Source: Cell Biol Toxicol. 2024 Sep 18;40(1):80. doi: 10.1007/s10565-024-09915-z (PMC11410999; doi:10.1007/s10565-024-09915-z)
Supplement: Supplementary file 1 — Supplementary file1 (PDF 16388 KB) [file 10565_2024_9915_MOESM1_ESM.pdf]

Supplementary Materials and Methods

Genotyping for wild-type and hepsin$^{-/-}$ mice.

The hepsin$^{-/-}$ mice were generated using traditional knockout techniques via homologous recombination in embryonic stem cells. A targeting vector was used to delete exons 9 and 10 of the hepsin gene, which encode the histidine and aspartic acid residues of the catalytic triad in serine proteases. This deletion was intended to result in functionally inactive hepsin, a hypothesis confirmed by the absence of hepsin transcript and protein, as reported in our previous publication (Yu et al. 2000). We have published several studies utilizing this well-established hepsin-/- mouse line (Hsu et al. 2012; Hsu et al. 2021).

In this study, the hepsin$^{-/-}$ mice were obtained through heterozygous inbreeding, as detailed in the Materials and Methods section and identified via genotyping PCR. The genomic DNA from wild-type mice was detected using specific primers Bstx1 (5'-AGG AAG CTG CCG GTG GAC CGC ATT-3') and mouse hep 6D (5'-CCG AGA CAG GAC CCG GTT CC-3'), resulting in a PCR product of 300 bp. Since Hepsin$^{-/-}$ mice were generated by replacing exons 9 and 10 with a neomycin (Neo) cassette, the hepsin$^{-/-}$ allele was detected using the primers Bstx1 and Neo5U (5'-AAC TGT TCG CCA GGC TCA AGG C-3'), yielding a PCR product of 900 bp due to the Neo cassette, as shown in the illustration and PCR results in Fig. S1.

Biochemistry tests

Serum samples were prepared from blood collected via heart puncture and analyzed using a Fuji Dri-Chem 4000i to assess the concentration of aspartate transaminase (AST) and alanine transaminase (ALT).

Analysis of degeneration area in mouse liver

The liver of each mouse, embedded in paraffin, was sectioned into 5-μm-thick slices and stained with hematoxylin and eosin. Regions displaying degeneration, characterized by cytoplasmic vacuolation in hepatocytes, were specifically selected for area quantification using ImageJ software (NIH). The data, calculated from 10 fields per mouse, are presented as the average percentage of the degeneration area relative to the total field area observed at 100× magnification.

Detection of APAP-cysteine

To detect APAP-cysteine, liver lysates were prepared as described before (Frey et al. 2015). Briefly, approximately 100 mg of mouse liver tissue was lysed in 500 μl of 10 mM sodium acetate buffer (pH 6.5) using a high-speed shaker (TissueLyser II, QIAGEN). The lysate was then centrifuged at 16,000 × g for 20 minutes at 4°C. The

supernatants were collected and filtered through Bio-Spin 6 columns (Bio-Rad, Hercules, CA) for subsequent analysis with the BCA Protein Assay™ kit (Pierce) and for 24-hour digestion with protease type XIV from Streptomyces griseus (8 U/ml) (Sigma, P5147) at 50°C.

Each supernatant was then diluted 1:2 with 40% trichloroacetic acid to precipitate residual protein on ice for 10 minutes. After centrifugation at 16,000 × g for 10 minutes at 4°C, the supernatant was filtered through a 0.2-μm polytetrafluoroethylene membrane and immediately analyzed by ultra-performance liquid chromatography-tandem mass spectrometry (ExionLC™-AB SCIEX 4500) provided by TRI NEO BIOTECHNOLOGY. The standard curve for APAP-cysteine ranged from 0 to 100 ng/ml (ppb) by 2-fold serial dilution using 3-cysteinylacetaminophen trifluoroacetic acid salt (CAS 1331891-93-0) from Santa Cruz Biotechnology (sc-209586).

Analysis of total GSH content

Total GSH content in mouse liver was assayed using the Glutathione Assay Kit (Cayman, 703002). Briefly, approximately 100 mg of mouse liver tissue was lysed in 500 μl of 50 mM MES buffer (pH 6–7, containing 1 mM EDTA) using a TissueLyser II. The lysate was then centrifuged at 10,000 × g at 4°C for 15 minutes, and the supernatants were collected into new 1.5-ml Eppendorf tubes and kept on ice. Each

supernatant was diluted with an equal volume of MPA Reagent (prepared by dissolving 5 g of metaphosphoric acid in 50 ml sterile double-distilled water; Sigma-Aldrich, 239275), followed by vigorous mixing and a 5-minute incubation at room temperature. After centrifugation at 3000 × g at 4°C for 5 minutes, the supernatants were transferred to new 1.5-ml Eppendorf tubes and stored at –20°C for subsequent total GSH assay.

For the assay, 100 µl of supernatant from either the APAP treatment group or the control group was used. The control group sample was diluted 1:100 with GSH MES Buffer (Cayman, 703010). Next, 5 µl of TEAM Reagent (4 M solution of triethanolamine, Sigma-Aldrich, T58300) was added to each prepared sample, and 50 µl of this mixture was then added to 150 µl of freshly prepared assay cocktail (Cayman, 703002). After 25 minutes of incubation at room temperature in the dark, the optical density at 405 nm was measured for each sample.

Liver lysate preparation and immunoblotting

Approximately 100 mg of mouse liver tissue was lysed in RIPA buffer (50 mM Tris-Cl pH 7.5, 150 mM NaCl, 1% (v/v) NP-40 (CA630), 0.5% (w/v) sodium deoxycholate, 0.1% (v/v) SDS, 1 mM EDTA) with freshly added 1% (v/v) protease inhibitor, 1 mM phenylmethylsulfonyl fluoride, 1 mM DL-dithiothreitol (Sigma, D9163), and 1–2× PhosSTOP™ working solution (Sigma, 4906837001). The liver

tissue was then shaken at high speed using a TissueLyser II (QIAGEN), and the mixture was centrifuged at 16,000 × g for 20 minutes at 4°C. The supernatants (liver lysates) were stored at –80°C for subsequent BCA assay (Pierce BCA Protein Assay kit) and immunoblotting.

Each mouse liver lysate was mixed with 3× SDS-PAGE loading buffer, and 50 µg of total protein was loaded per lane for analysis by SDS-PAGE. Proteins were electrotransferred to a polyvinylidene difluoride membrane at 100 V for 90 minutes at 4°C. The membrane was then blocked with Tris-buffered saline containing 1% (v/v) Triton X-100 and 5% fat-free milk for 0.5–1 hour. It was incubated overnight at 4°C with primary antibodies at 1:1000 dilution: anti-hepsin (Abcam, ab31148), anti-nitrotyrosine (Abcam, ab7048), anti-cytochrome P450 2E1 (Abcam, ab28146), anti-cytochrome P450 1A2 (Abcam, ab22717), anti-RIP1 (Abcam, ab72139), anti-RIP3 (Cell Signaling, #15828), and anti-α-tubulin (Cell Signaling, #2144). The membrane was then incubated at room temperature for 1 hour with anti-rabbit or anti-mouse IgG-conjugated horseradish peroxidase (1:5000) as the secondary antibody. Images of the immunoblots were captured using an infrared camera (LAS-4000, Fujifilm).

Analysis of CYP1A2 activity in mouse liver

CYP1A2 activity in mouse liver lysates was assayed with a CYP1A2 Activity

Assay kit (fluorometric; Abcam, ab211074).

Transcriptome analysis of mouse liver after APAP treatment

Mouse liver tissue was lysed and homogenized using the liquid nitrogen grounding method for subsequent RNA extraction with the RNeasy Mini kit (QIAGEN, 74104). Prepared RNA was quantified with a NanoDrop spectrophotometer (Thermo Fisher Scientific), and a preliminarily check of RNA quality was conducted using 1% agarose electrophoresis to ensure distinct 28S and 18S bands.

RNA sequencing was conducted with the Illumina platform. Using a significance level of posterior probability of equal expression (PPEE) < 0.05 as the selection criteria, the selected differentially expressed genes at 1 and 2 hours after 400 mg/kg APAP treatment in hepsin$^{-/-}$ mouse liver compared with wild-type mouse liver were identified through analysis of Venn diagrams, clustering, and functional enrichment based on KEGG and WikiPathways using the DAVID platform. Gene set enrichment analysis was conducted with the entire transcriptome dataset. Results from all the aforementioned analyses were plotted using http://www.bioinformatics.com.cn/srplot.

Immunofluorescence staining

Cryopreserved liver tissue was sectioned into 8-μm-thick slices for

immunofluorescence staining. Primary antibodies used were anti-Cx32 (Invitrogen, 710600) and anti-glutamine synthetase (BD, 610517). Goat anti-rabbit Cy3 and goat anti-mouse Alexa 488 were used for the detection of Cx32 and glutamine synthetase, respectively. Immunofluorescence staining was observed using an Olympus BX63 fluorescence microscope. Confocal laser-scanning microscopy was performed with a Zeiss LSM780 microscope. Fluorescence intensity was quantified for 5–10 fields from each mouse at 100× magnification using ImageJ software.

Incision loading/dye transfer in mouse liver for functional assay of GJ communication

Mice were treated with 1 mg/kg 2-APB for 3 hours, after which they were sacrificed, and the liver was removed for incision loading/dye transfer following a procedure described in previous studies (Patel et al. 2012; Sai et al. 2000). Briefly, the left lobe of the liver was excised and filled with a fluorescent dye mixture consisting of 0.5% Lucifer yellow (Biotium, 80015) and 0.5% rhodamine-dextran (molecular mass 10,000 Da; Invitrogen, D1863) suspended in PBS. Three incisions, each measuring 7–8 mm in length and 1 mm in depth, were made on the surface of each liver specimen using a sharp blade. The dye mixture was applied to these incisions and incubated for 5 minutes at room temperature.

Subsequently, each liver was washed three times with PBS and then fixed in 10% formalin overnight. After cryopreservation, 8-μm-thick sections were obtained perpendicular to the incision line. These sections were examined using a fluorescence microscope at a magnification of 200×. Areas stained with Lucifer yellow but lacking rhodamine-dextran staining were carefully identified. Using ImageJ software, the distance perpendicular to the incision line within these stained areas was measured. For each incision, 20 random measurement sites were selected, and the data represent the average of 60 measurements per mouse.

Staining for ROS with dihydroethidium

Mice were treated with 500 mg/kg thioacetamide for 6 hours and then sacrificed. The left lobe of each liver was immediately frozen in liquid nitrogen for cryopreservation. DHE staining was conducted as quickly as possible, with several precautions taken to minimize potential ROS degradation (Patel et al. 2012; Solsona-Vilarrasa et al. 2019; Zhan et al. 2019; Zheng et al. 2020). Liver tissues were immediately cryopreserved in liquid nitrogen as a cryoblock in O.C.T. compound, and freshly cut frozen liver sections (6 μm) were prepared without delay. Following a brief rinse with PBS, the liver sections were stained with freshly prepared 5 μM

dihydroethidium (Invitrogen™, D1168) for 30 minutes at 37°C. After washing three times with PBS (5 minutes each), DAPI (100 µg/ml stock, diluted 1:100 in PBS as a working solution) was applied for 10 minutes at room temperature. The slides were then mounted and imaged using a fluorescence microscope (BX63, Olympus).

References

Frey SM, Wiegand TJ, Green JL, Heard KJ, Wilkins DG, Gorodetsky RM, Dart RC. Confirming the Causative Role of Acetaminophen in Indeterminate Acute Liver Failure Using Acetaminophen-Cysteine Adducts. J Med Toxicol. 2015;11:218-22. https://doi.org/10.1007/s13181-015-0476-x

Hsu YC, Huang HP, Yu IS, Su KY, Lin SR, Lin WC, Wu HL, Shi GY, Tao MH, Kao CH, Wu YM, Martin PE, Lin SY, Yang PC, Lin SW. Serine protease hepsin regulates hepatocyte size and hemodynamic retention of tumor cells by hepatocyte growth factor signaling in mice. Hepatology (Baltimore, Md). 2012;56:1913-23. https://doi.org/10.1002/hep.25773

Hsu YC, Yu IS, Tsai YF, Wu YM, Chen YT, Sheu JC, Lin SW. A Preconditioning Strategy to Augment Retention and Engraftment Rate of Donor Cells During Hepatocyte Transplantation. Transplantation. 2021;105:785-95. https://doi.org/10.1097/TP.0000000000003461

Patel SJ, Milwid JM, King KR, Bohr S, Iracheta-Vellve A, Li M, Vitalo A, Parekkadan B, Jindal R, Yarmush ML. Gap junction inhibition prevents drug-induced liver toxicity and fulminant hepatic failure. Nature biotechnology. 2012;30:179-83. https://doi.org/10.1038/nbt.2089

Solsona-Vilarrasa E, Fucho R, Torres S, Nunez S, Nuno-Lambarri N, Enrich C, Garcia-Ruiz C, Fernandez-Checa JC. Cholesterol enrichment in liver mitochondria impairs oxidative phosphorylation and disrupts the assembly of respiratory supercomplexes. Redox Biol. 2019;24:101214. https://doi.org/10.1016/j.redox.2019.101214

Yu IS, Chen HJ, Lee YS, Huang PH, Lin SR, Tsai TW, Lin SW. Mice deficient in hepsin, a serine protease, exhibit normal embryogenesis and unchanged hepatocyte regeneration ability. Thrombosis and haemostasis. 2000;84:865-70.

Zhan F, Zhao G, Li X, Yang S, Yang W, Zhou S, Zhang F. Inositol-requiring enzyme 1

alpha endoribonuclease specific inhibitor STF-083010 protects the liver from thioacetamide-induced oxidative stress, inflammation and injury by triggering hepatocyte autophagy. Int Immunopharmacol. 2019;73:261-9. https://doi.org/10.1016/j.intimp.2019.04.051

Zheng J, Chen L, Lu T, Zhang Y, Sui X, Li Y, Huang X, He L, Cai J, Zhou C, Liang J, Chen G, Yao J, Yang Y. MSCs ameliorate hepatocellular apoptosis mediated by PINK1-dependent mitophagy in liver ischemia/reperfusion injury through AMPKalpha activation. Cell death & disease. 2020;11:256. https://doi.org/10.1038/s41419-020-2424-1

Supplementary Figure Legends

Fig. S1. Genotyping for wild-type and hepsin$^{-/-}$ mice. (A) The diagram illustrates the genotyping primers used to identify wild-type (hepsin WT) and hepsin$^{-/-}$ (hepsin KO) mice. Genomic DNA for the wild-type allele was detected using the specific primers Bstx1 and mouse hep 6D, resulting in a 300 bp PCR product. In contrast, the hepsin$^{-/-}$ allele was detected using the primers Bstx1 and Neo5U, yielding a 900 bp PCR product due to the Neo cassette. (B) The genotyping PCR results show the genomic DNA of wild-type (WT) and hepsin$^{-/-}$ (KO) mice, using WT primers (Bstx1 and mouse hep 6D) and KO primers (Bstx1 and Neo5U), respectively. The PCR product sizes were as expected.

Fig. S2. Endogenous Mouse Hepsin Levels and Histology in the Early Stage After 400 mg/kg APAP Treatment. (A) Histological analysis at 0.5 and 1 hour post-APAP treatment. Images are shown at 100x magnification (scale bar: 200 μm) and 400x magnification (scale bar: 50 μm). (B) Immunoblotting of endogenous mouse hepsin in liver tissue at the indicated time points following APAP treatment (n=6 per group). Data are presented as the mean ± SD in bar charts, with significance levels denoted by asterisks: ***$p < 0.001$.

Fig. S3. Immunoblotting for cytochrome P450 2E1 (CYP2E1) and **cytochrome P450 1A2 (CYP1A2) and analysis activity of CYP1A2 Post-APAP Treatment.** (A) Immunoblotting and quantification of CYP2E1 and CYP1A2 in liver tissues at various time points following the administration of 400 mg/kg APAP (n = 6-12 per group). (B) Cytochrome P450 1A2 activity in each mouse liver at 1 and 2 hours post-treatment with 400 mg/kg APAP (n = 9-14 per group). NS indicates the normal saline control group. Data are presented as mean ± SD in bar charts.

Fig. S4. Heatmap displaying the transcriptome of the PI3K-Akt and mTOR signaling pathways. (A)Transcriptome of the PI3K-Akt signaling pathway showing a significant difference (p = 0.049) between the normal saline control (NS) group and the 1-hour post-APAP treatment group in wild-type mice (n = 2-3 per group). (B) Transcriptome of the mTOR signaling pathway showing significant differences (p = 0.033 and p =0.021) between the normal saline group and the 1-hour or 2-hour post-APAP treatment groups in wide-type mice, respectively (n = 2-3 per group).

Fig. S5. Heatmap displaying the transcriptome of the GJ pathway. The transcriptome of the GJ pathway showed a significant difference (p = 0.0021) between

wild-type and hepsin$^{-/-}$ mice at 1-hour post-APAP treatment (n = 2-3 per group).

Fig. S6. Change in Cx32 expression levels in the liver between hepsin$^{-/-}$ and wild-type mice after APAP insult. Representative immunofluorescence photomicrographs of mouse liver sections stained for Cx32, at the indicated time points after treatment with 400 mg/kg APAP. NS, normal saline control group (n = 4-8 per group). The quantified relative fluorescence intensity of Cx32 in each group is shown in Figure 4A.

Fig. S7. APAP induces hepatotoxicity in wild-type and hepsin$^{-/-}$ mice under non-fasting conditions. Survival curves of non-fasting wild-type (WT) and hepsin$^{-/-}$ (KO) mice after (A) 300 mg/kg and (B) 600 mg/kg APAP treatment. Sample sizes for each group are indicated in brackets, and statistical significance was determined using the log-rank test.

Fig. S8. Levels and heatmap of the transcriptome for glutathione (GSH) biosynthesis-related genes. (A) The bar chart shows the transcriptome levels from RNA transcriptome analysis of genes involved in the GSH biosynthesis pathway. No significant differences were observed between wild-type (WT) and hepsin$^{-/-}$ (KO) mice in either the normal saline control (NS) or post-APAP treatment groups at 1 hour and 2

hours. (B) The heatmap presents the relative transcriptome levels of the genes involved in the GSH biosynthesis pathway in the normal saline group and the post-APAP treatment treatment groups at 1 hour and 2 hours. (n = 2-3 per group). *Gclc*, Glutamate-cysteine ligase. *Gclm,* Glutamate-cysteine ligase. *Gss,* Glutathione synthetase.

Fig. S9. Levels and heatmap of the mitochondrial-related genes. (A) The bar chart shows the transcriptome levels from RNA transcriptome analysis of mitochondrial genes, including *Cox7a1*, *Cpt2*, and *Cpt1b*. No significant differences were observed between wild-type (WT) and hepsin$^{-/-}$ (KO) mice in either the normal saline control (NS) or post-APAP treatment groups at 1 hour and 2 hours. (B) The heatmap presents the relative transcriptome levels of general mitochondrial-related genes in the normal saline group and the post-APAP treatment groups at 1 hour and 2 hours (n = 2-3 per group). *Cox7a1*, Cytochrome c oxidase. *Cpt2*, Carnitine palmitoyltransferase 2. *Cpt1b*, Carnitine palmitoyltransferase 1B.

Fig. S10. The abundance of necroptosis markers RIP1 and RIP3 after APAP treatment. Immunoblotting and quantification of RIP1 and RIP3 in wild-type (WT) and hepsin$^{-/-}$ (KO) mouse liver at the indicated time points post-treatment with 400 mg/kg APAP (n = 5-11 per group). Data are presented as mean ± SD in bar charts. No

significant differences were observed between wild-type and hepsin$^{-/-}$ mice in either the normal saline control (NS) or post-APAP treatment groups at any time point.

A

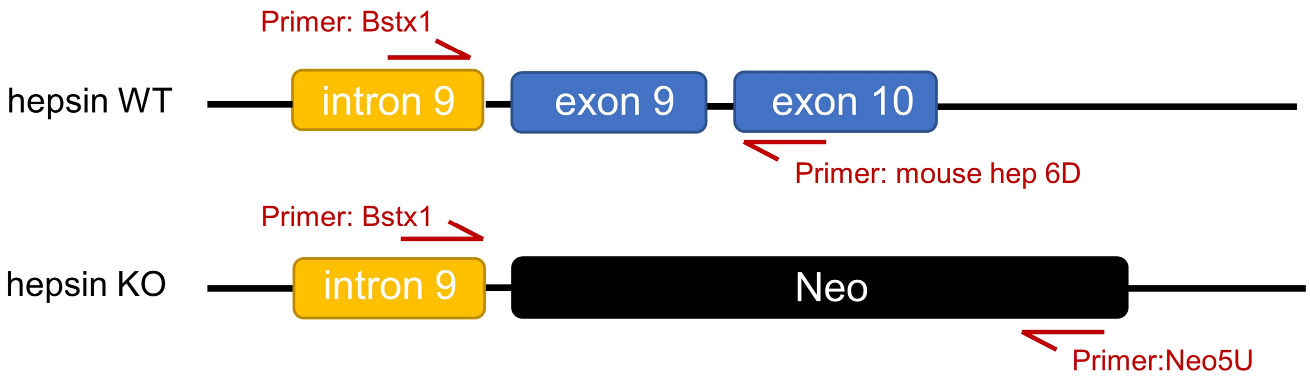
Primer: Bstx1
hepsin WT
intron 9
exon 9
exon 10
Primer: mouse hep 6D
Primer: Bstx1
hepsin KO
intron 9
Neo
Primer:Neo5U

B

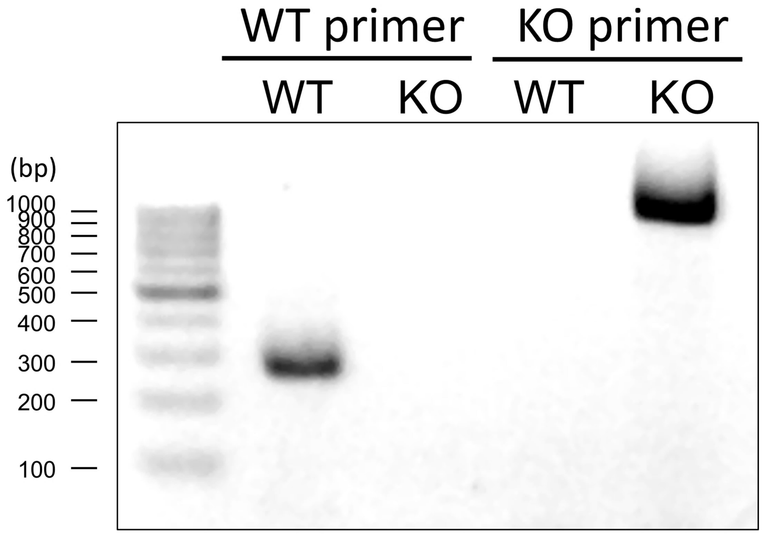
WT primer
KO primer
WT
KO
WT
KO
(bp)
1000
900
800
700
600
500
400
300
200
100

Fig. S1

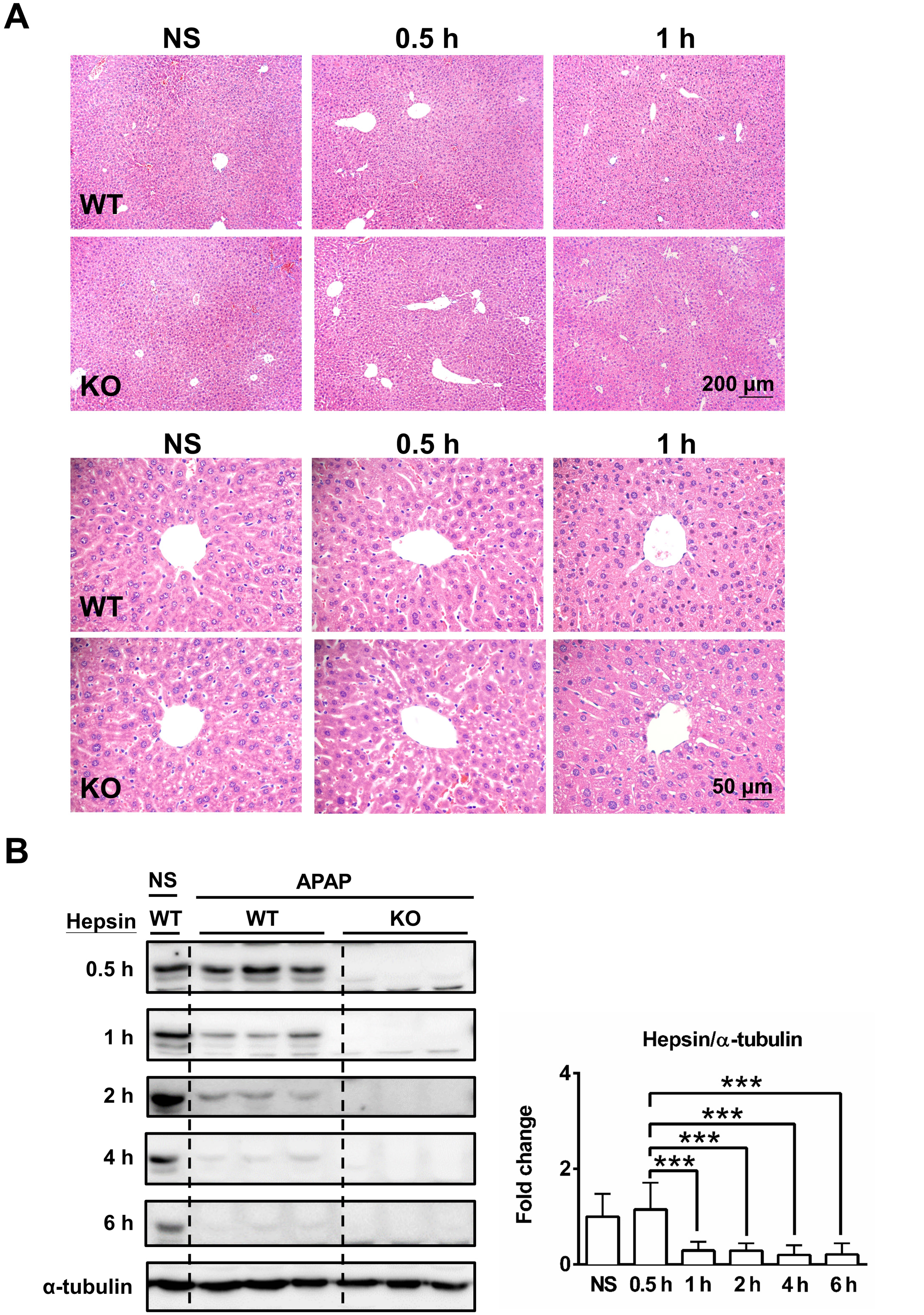
A
NS
0.5 h
1 h
WT
KO
200 µm
50 µm
B
NS
APAP
Hepsin
WT
WT
KO
0.5 h
1 h
2 h
4 h
6 h
α-tubulin
Hepsin/α-tubulin
Fold change
4
2
0
***
***
***
***
NS
0.5 h
1 h
2 h
4 h
6 h

Fig. S2

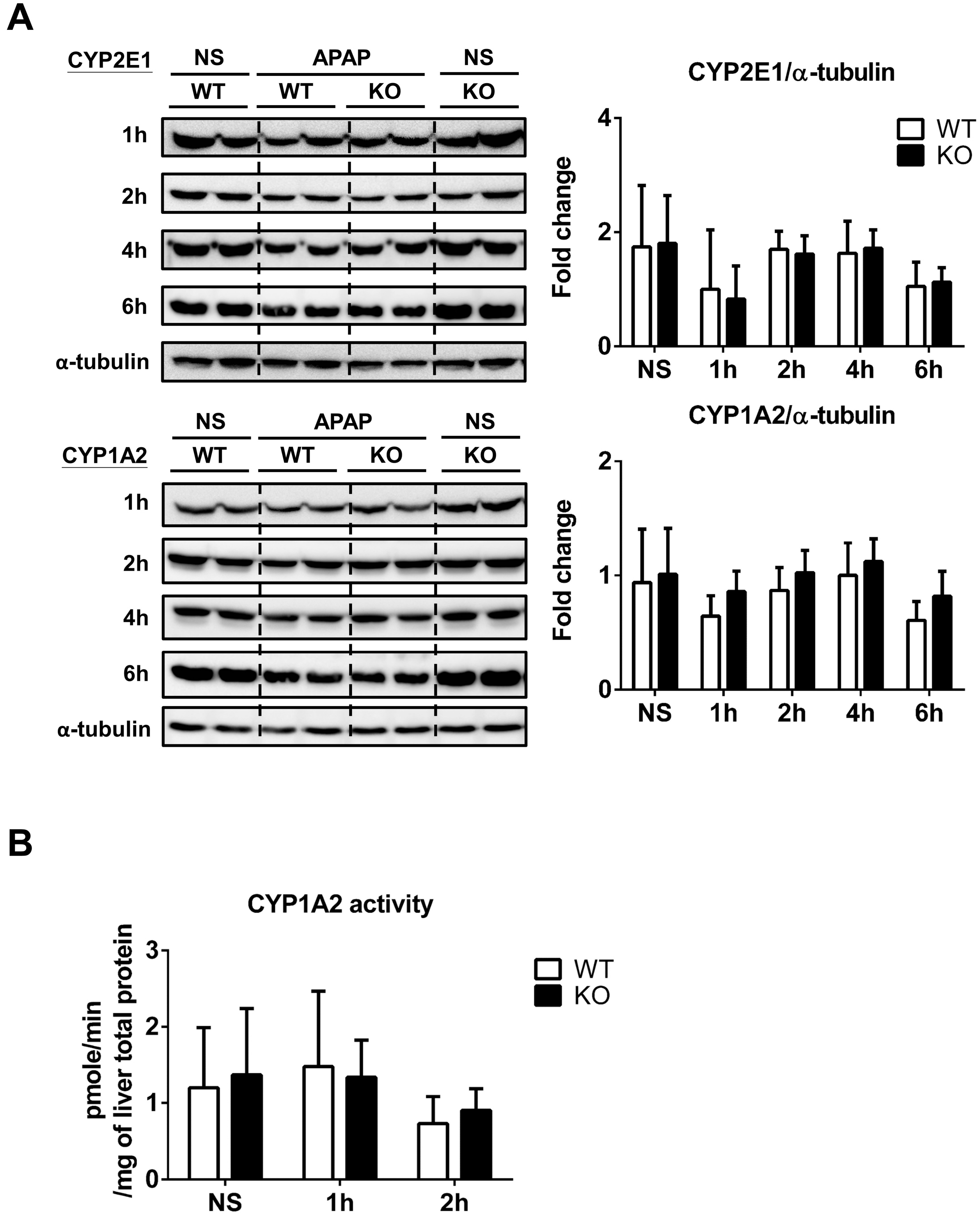

A
CYP2E1
NS
APAP
NS
WT
WT
KO
KO
1h
2h
4h
6h
α-tubulin
CYP2E1/α-tubulin
Fold change
WT
KO
NS
1h
2h
4h
6h
NS
APAP
NS
CYP1A2
WT
WT
KO
KO
1h
2h
4h
6h
α-tubulin
CYP1A2/α-tubulin
Fold change
NS
1h
2h
4h
6h
B
CYP1A2 activity
pmole/min
/mg of liver total protein
WT
KO
NS
1h
2h

Fig. S3

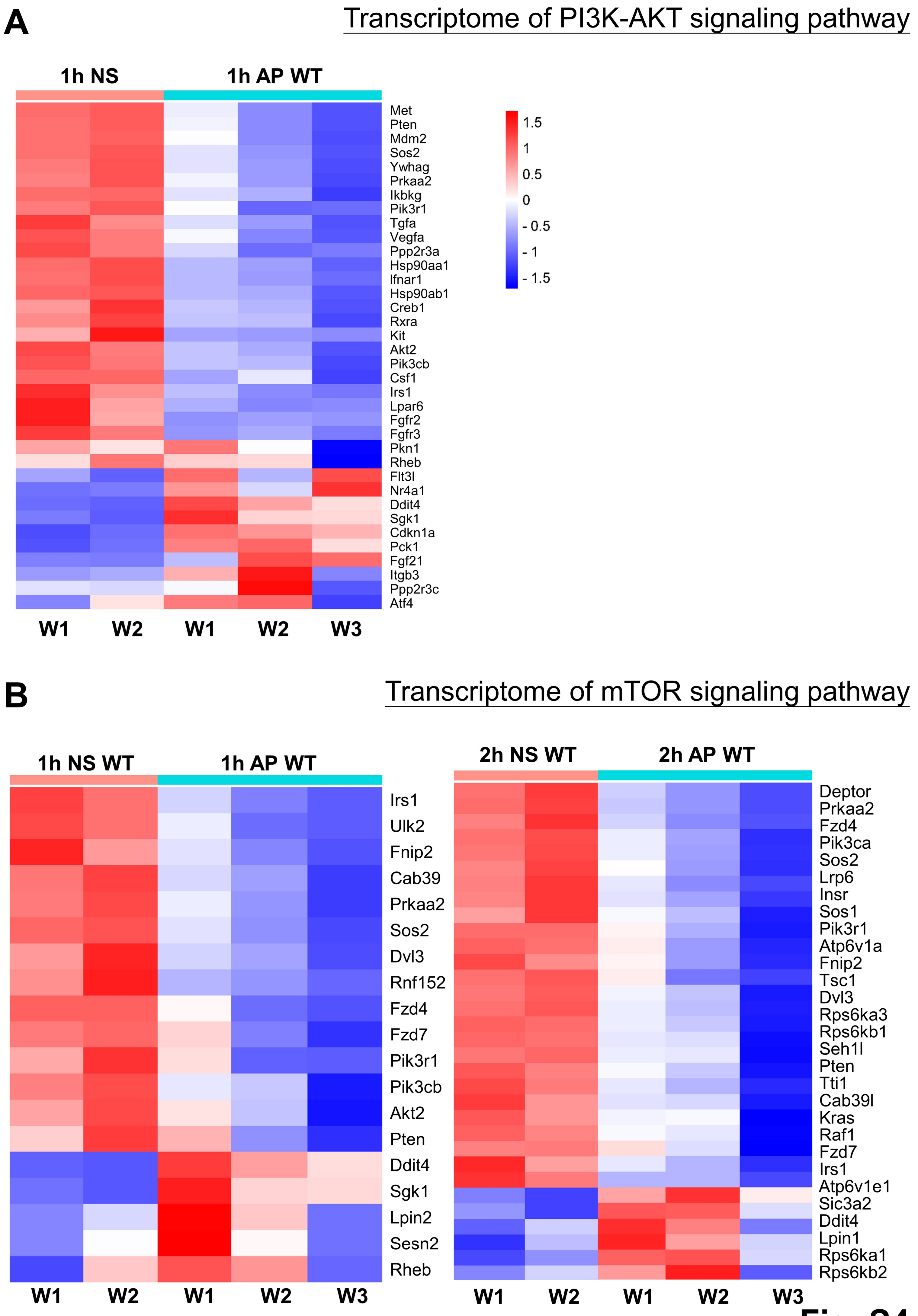
A
Transcriptome of PI3K-AKT signaling pathway
1h NS
1h AP WT
Met
Pten
Mdm2
Sos2
Ywhag
Prkaa2
Ikbkg
Pik3r1
Tgfa
Vegfa
Ppp2r3a
Hsp90aa1
Ifnar1
Hsp90ab1
Creb1
Rxra
Kit
Akt2
Pik3cb
Csf1
Irs1
Lpar6
Fgfr2
Fgfr3
Pkn1
Rheb
Flt3l
Nr4a1
Ddit4
Sgk1
Cdkn1a
Pck1
Fgf21
Itgb3
Ppp2r3c
Atf4
1.5
1
0.5
0
- 0.5
- 1
- 1.5
W1
W2
W1
W2
W3
B
Transcriptome of mTOR signaling pathway
1h NS WT
1h AP WT
Irs1
Ulk2
Fnip2
Cab39
Prkaa2
Sos2
Dvl3
Rnf152
Fzd4
Fzd7
Pik3r1
Pik3cb
Akt2
Pten
Ddit4
Sgk1
Lpin2
Sesn2
Rheb
W1
W2
W1
W2
W3
2h NS WT
2h AP WT
Deptor
Prkaa2
Fzd4
Pik3ca
Sos2
Lrp6
Insr
Sos1
Pik3r1
Atp6v1a
Fnip2
Tsc1
Dvl3
Rps6ka3
Rps6kb1
Seh1l
Pten
Tti1
Cab39l
Kras
Raf1
Fzd7
Irs1
Atp6v1e1
Slc3a2
Ddit4
Lpin1
Rps6ka1
Rps6kb2
W1
W2
W1
W2
W3

Fig. S4

Transcriptome of GJ pathway

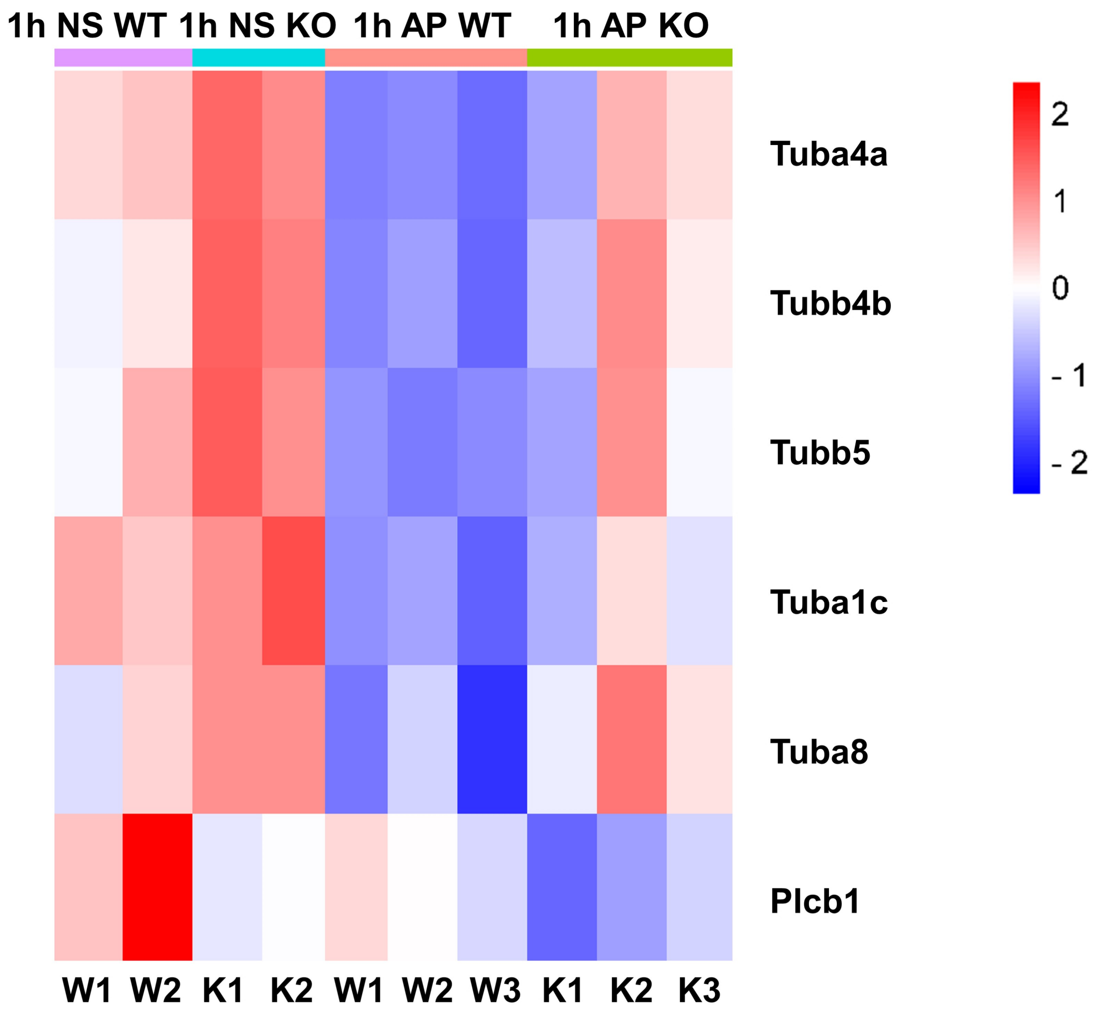

Fig. S5

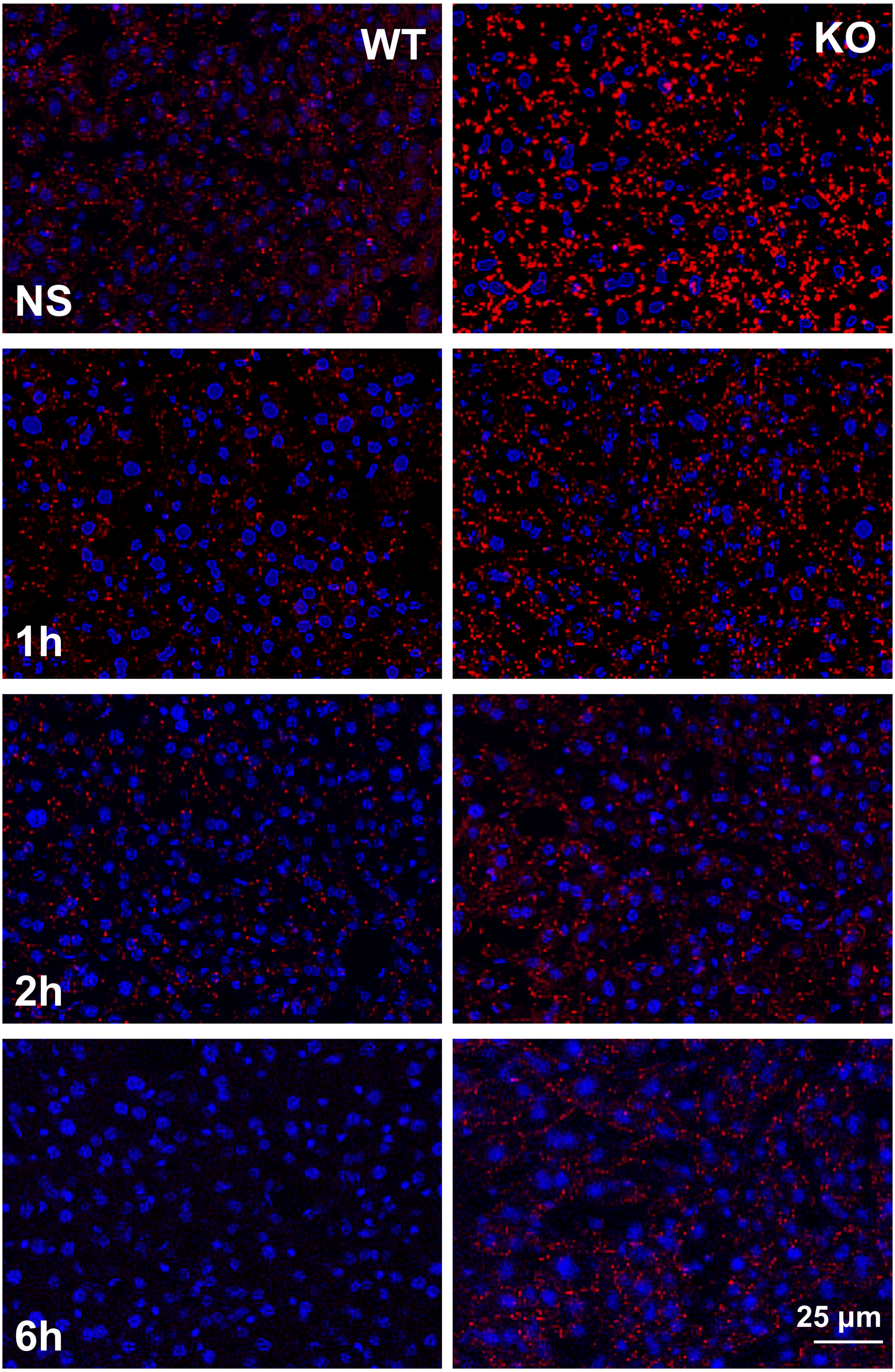

Fig. S6

A

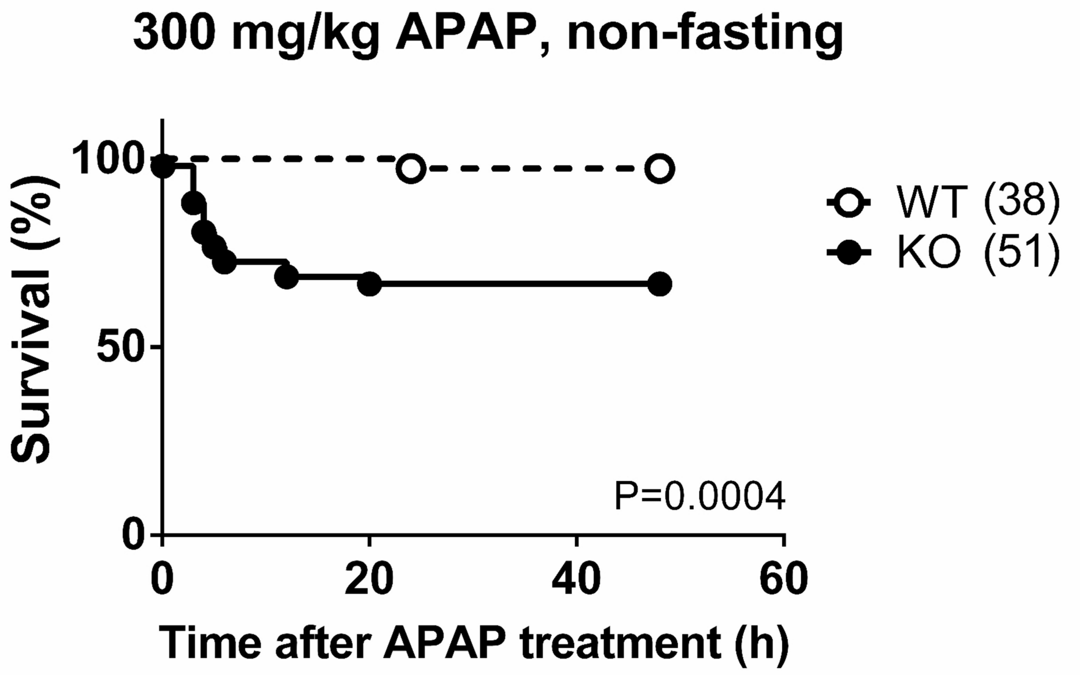
300 mg/kg APAP, non-fasting
Survival (%)
100
50
0
0
20
40
60
Time after APAP treatment (h)
WT (38)
KO (51)
P=0.0004

B

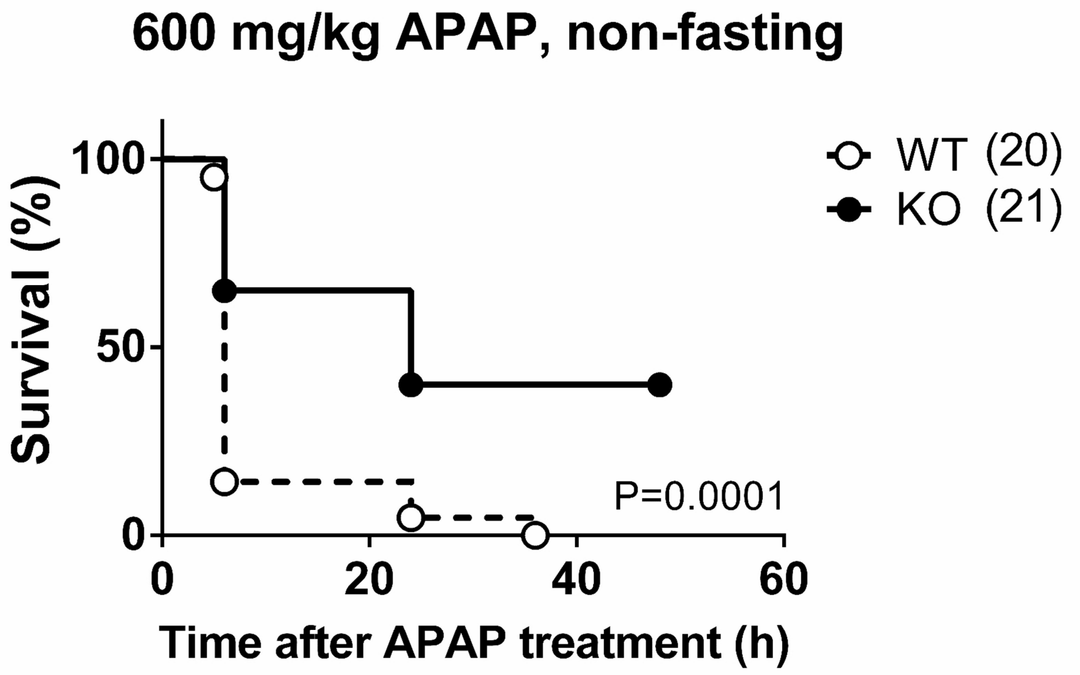
600 mg/kg APAP, non-fasting
Survival (%)
100
50
0
0
20
40
60
Time after APAP treatment (h)
WT (20)
KO (21)
P=0.0001

Fig. S7

A Transcriptome of GSH biosynthesis-related genes

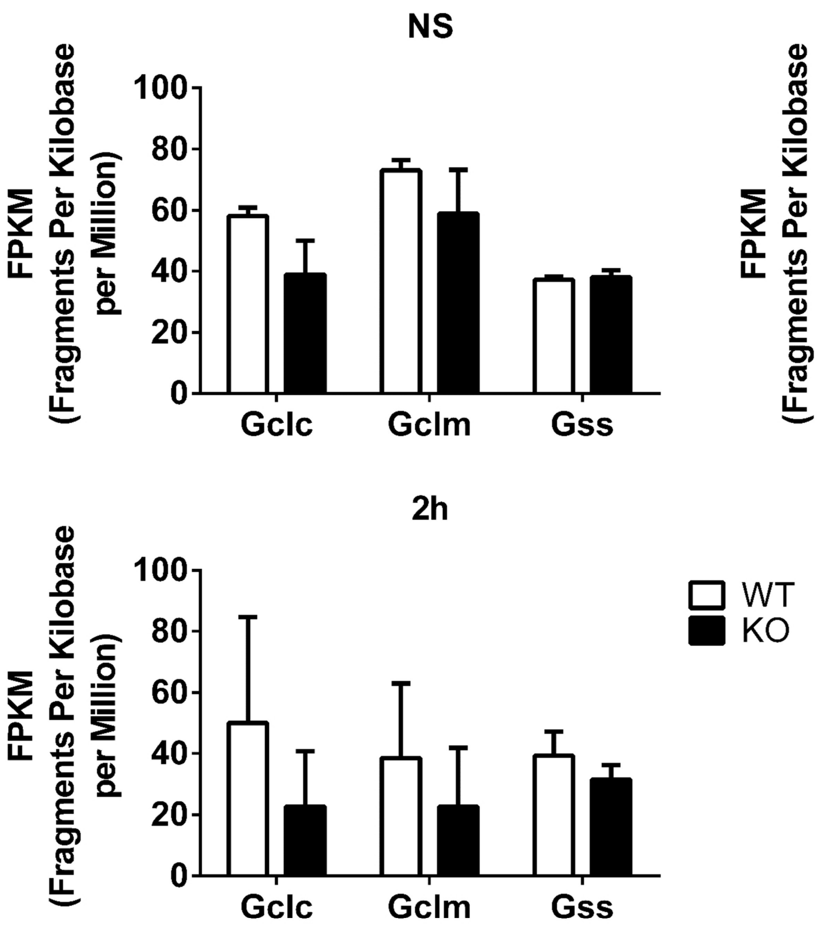

B

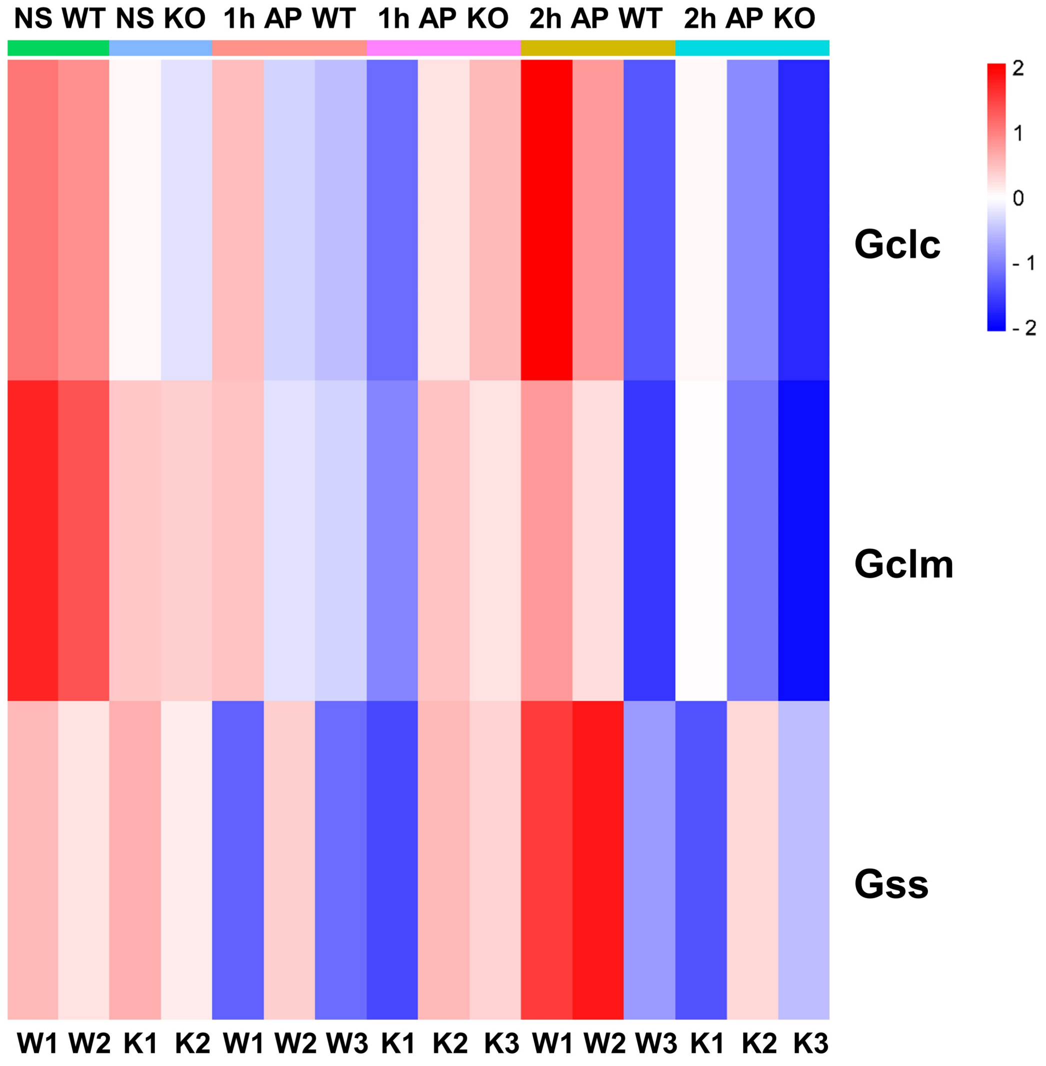

Fig. S8

A Transcriptome of Mitochondrial-related genes

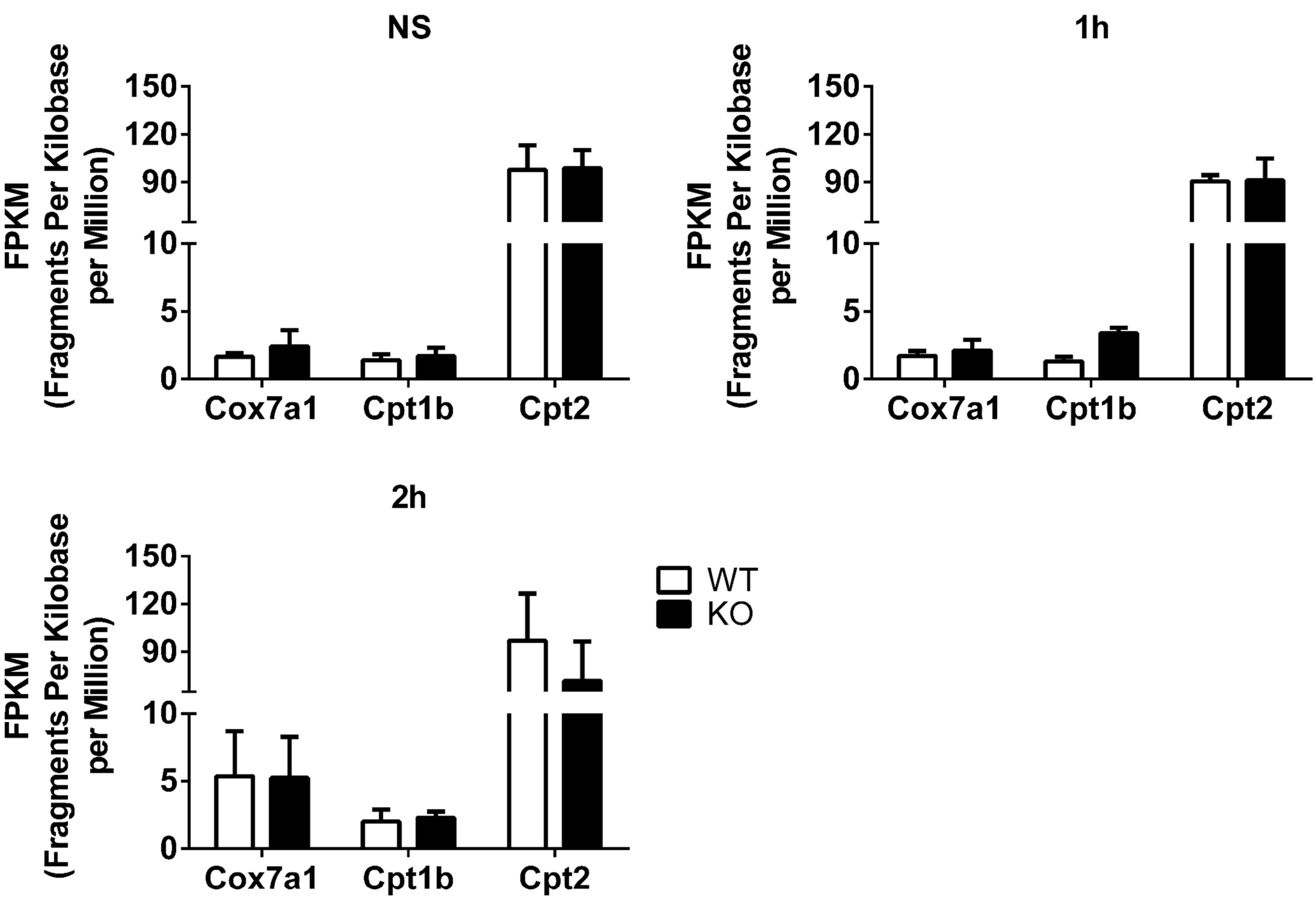

B

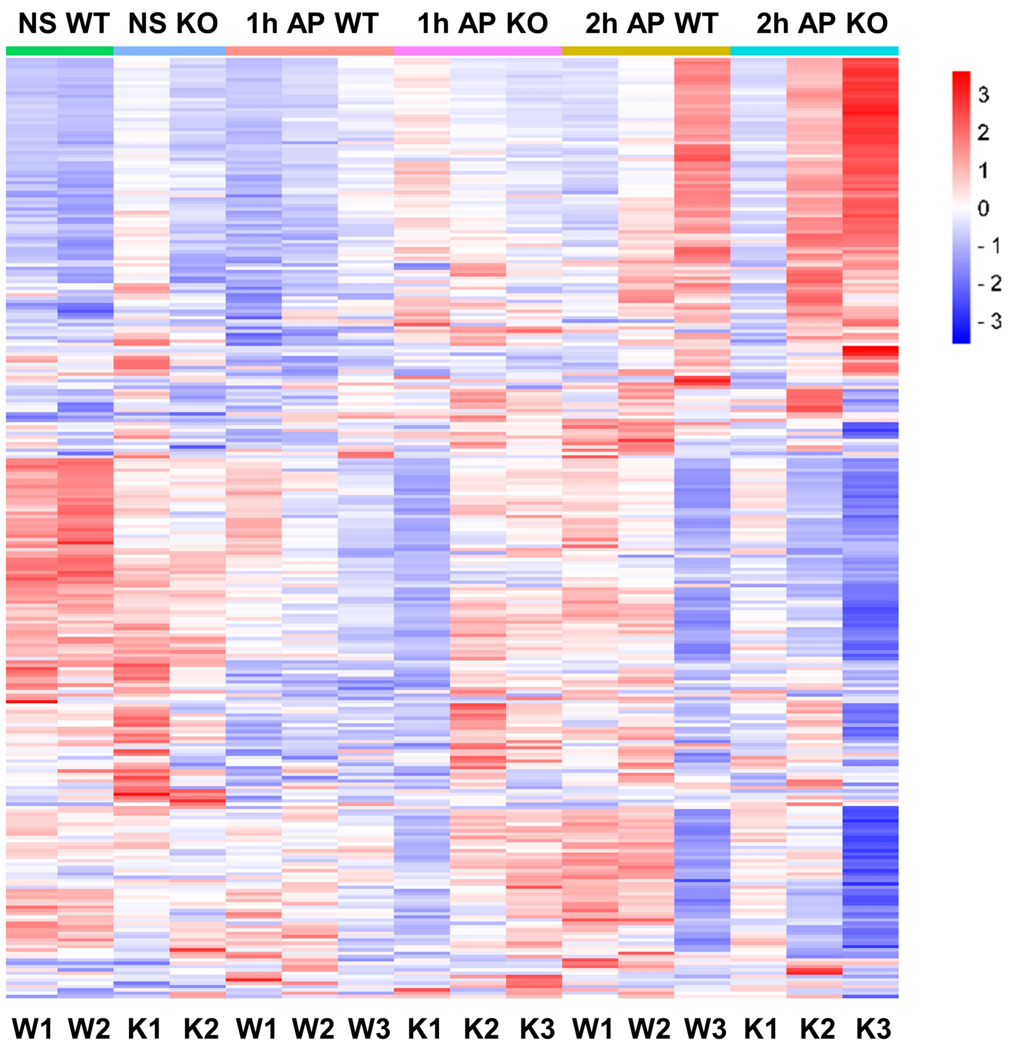

Fig. S9

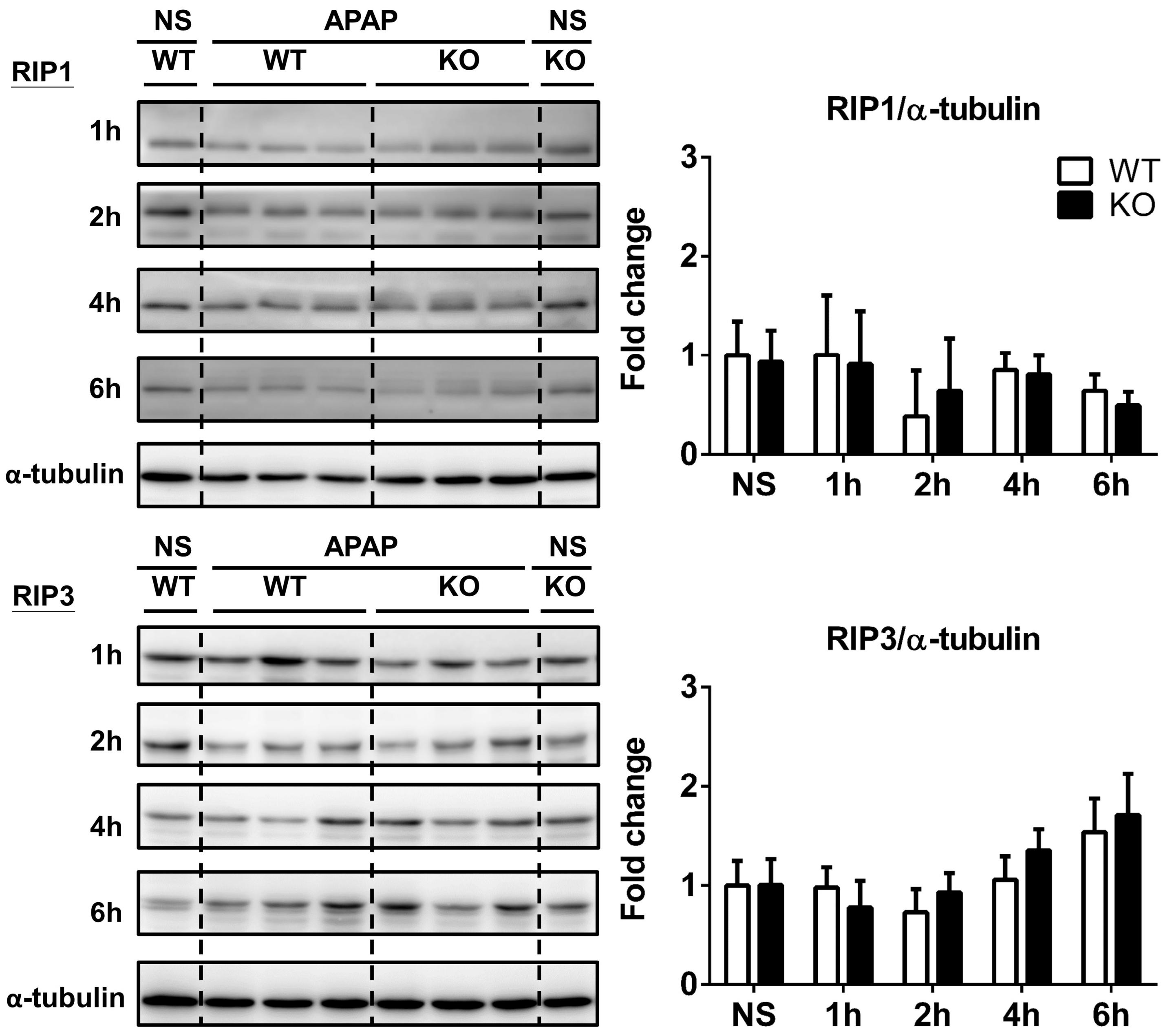

RIP1
NS
APAP
NS
WT
WT
KO
KO
1h
2h
4h
6h
α-tubulin
RIP1/α-tubulin
WT
KO
Fold change
0
1
2
3
NS
1h
2h
4h
6h
RIP3
NS
APAP
NS
WT
WT
KO
KO
1h
2h
4h
6h
α-tubulin
RIP3/α-tubulin
Fold change
0
1
2
3
NS
1h
2h
4h
6h

Fig. S10
